# Supplementary material for: Fertility-sparing surgery with neoadjuvant chemotherapy in early and locally advanced cervical cancer: A clinical protocol
Source: PLoS One. 2026 Jan 13;21(1):e0340963. doi: 10.1371/journal.pone.0340963 (PMC12798975; doi:10.1371/journal.pone.0340963)
Supplement: S2 File — (DOCX) [file pone.0340963.s002.docx]

**妊孕性温存を希望する子宮頸癌IB2-IB3期に対する**

**主治療前化学療法を用いた縮小手術**

Fertility preservation using neoadjuvant chemotherapy

and cervical conization followed by laparoscopic pelvic lymphadenectomy for　FIGO stage IB2-IB3 cervical cancer

**（CC-NAC C: FepCC）**

研究計画書

【統括管理者】

長尾　昌二

　岡山大学学術研究院医歯薬学域　周産期医療学講座

　住所：〒700-8558　岡山県岡山市北区鹿田町2-5-1

　電話番号：086-235-7320

2025年1月1日作成　第1.0版

2025年5月29日改訂　第1.1版

2025年6月17日改訂　第2.0版

2025年7月2日改訂　第3.0版

**0．研究の概要**

1. 目的

本研究の目的は、腫瘍径２cmを超える局所進行子宮頸癌（FIGO進行期IB2〜IB3期（2018年分類）の扁平上皮癌、腺癌、腺扁平上皮癌）に対して主治療前化学療法（Neoadjuvant chemotherapy：NAC）後に子宮頸部円錐切除術および腹腔鏡下骨盤リンパ節郭清術による妊孕性温存を行い、その腫瘍学的安全性及び周産期医学的・女性医学的妥当性を確認することである。

1. 主要評価項目

①子宮温存可能割合

ただし、化学放射線同時化学療法(Concurrent chemo radiotherapy：CRT)を実施した場合には子宮温存には含めない。

1. 副次評価項目
   1. 2年無再発生存割合
   2. 2年無再発生存割合
   3. 2年全生存割合
   4. 無再発生存期間
   5. 全生存期間
   6. 女性医学的予後（QOLを含む）
   7. 月経再開に至った割合・排卵再開に至った割合
   8. 妊娠に至った割合
   9. 生児獲得割合、流産・早産割合
   10. 毒性の種類、グレードおよび発生割合（CTCAE ver.5.0を用いる）

1. 対象患者

選択基準

① 臨床的に子宮頸癌ⅠB2あるいはⅠB3期と診断された患者（FIGO2018年分類）

② 病理組織学的に診断の確定した扁平上皮癌、腺癌、腺扁平上皮癌の患者

③ 未閉経の患者

④ 40歳以下の患者

⑤ 十分な全身の臓器機能を有する患者

⑥ 妊孕性温存を希望する患者

⑦ 本研究の参加にあたり十分な説明を受けた後、十分な理解の上、患者本人の自由意思による文書による同意が得られた患者

除外基準

1. HPV-independent癌の患者
2. 全ての活動性の重複癌患者
3. 重篤な合併症を有する患者
4. ポリオキシエチレンヒマシ油（クレモホールＥＬ^R^）含有製剤（シクロスポリンなど）および､硬化ヒマシ油含有製剤（注射用ビタミン剤など）の投与歴に関連して過敏症が発現したことのある患者
5. 抗菌剤を必要とする活動性の感染症患者
6. 妊娠､授乳中及び妊娠している可能性のある患者
7. その他、研究責任医師、研究分担医師が研究対象者として不適当と判断した患者
8. 介入内容
9. 研究実施期間

認定臨床研究審査委員会承認後のjRCT^＊^公表日から2030年3月31日まで

1. 目標症例数

10例

目次

[１．研究の名称 8](#_Toc197695254)

[２．研究の実施体制（実施医療機関の名称及び研究者等の氏名を含む） 8](#_Toc197695255)

[３．研究の背景、目的、意義 8](#_Toc197695256)

[４．研究の方法 10](#_Toc197695257)

[（１）研究の種類・デザイン 10](#_Toc197695258)

[（５）被験薬の管理方法 19](#_Toc197695259)

[（７）症例登録 19](#_Toc197695260)

[５．観察及び検査項目 20](#_Toc197695261)

[（１）研究対象者背景 20](#_Toc197695262)

[（２）検査項目 20](#_Toc197695263)

[（３）自他覚症状の確認 21](#_Toc197695264)

[（４）有害事象と副作用の確認（スクリーニング時、dose dense TC療法中、腹腔鏡下リンパ節郭清1ヶ月後まで） 21](#_Toc197695265)

[６．評価項目 22](#_Toc197695266)

[（１）主要評価項目（Primary endpoint） 22](#_Toc197695267)

[（２）副次評価項目（Secondary endpoint） 23](#_Toc197695268)

[７．目標症例数及び設定根拠 23](#_Toc197695269)

[８．データの集計及び統計解析方法 24](#_Toc197695270)

[９．研究実施期間 25](#_Toc197695271)

[１０．研究対象者の設定方針 25](#_Toc197695272)

[（２）除外基準 26](#_Toc197695273)

[１１．研究の背景及び科学的合理性の根拠（研究の合理性・妥当性） 26](#_Toc197695274)

[１２．インフォームド・コンセントを受ける手続き 29](#_Toc197695275)

[１３．個人情報等の取扱い（個人情報の加工の方法を含む） 30](#_Toc197695276)

[本研究に係わるすべての研究者は、「ヘルシンキ宣言」、「臨床研究法」並びに「個人情報保護法」を遵守して実施する。研究実施に係る試料・情報を取扱う際は、研究対象者に研究独自の研究用IDを割り振り、氏名と研究用IDとの対応表を作成する。元データからは氏名を削除し、研究対象者の秘密保護に十分配慮した上で研究に用いる。研究期間を通して対応表ファイルはパスワードをかけ、漏洩しないように厳重に保管する。研究の結果を公表する際は、氏名、生年月日などの直ちに研究対象者を特定できる情報を含まないようにする。また、研究の目的以外に、研究で得られた研究対象者の試料・情報を使用しない。 30](#_Toc197695277)

[１４．研究対象者に生じる負担、予測されるリスク及び利益、これらの総合的評価並びに当該負担及びリスクを最小化する対策 30](#_Toc197695278)

[１５．記録(データを含む)の取扱い及び保存・破棄の方法（保管期間を含む） 36](#_Toc197695279)

[１６．原資料等の閲覧に関する事項 37](#_Toc197695280)

[１７．定期報告 37](#_Toc197695281)

[１８．研究の資金源、研究期間の研究に係る利益相反及び個人の収益等、研究等の研究に係る利益相反に関する状況 37](#_Toc197695282)

[１９．研究に関する情報公開の方法（研究計画の登録及び研究結果の公表） 38](#_Toc197695283)

[２０．研究対象者等からの相談等への対応 38](#_Toc197695284)

[２１．代諾者等からのインフォームド・コンセントを受ける場合の手順 38](#_Toc197695285)

[２２．インフォームド・アセントを得る手続き（説明事項、説明方法含む） 38](#_Toc197695286)

[２３．緊急かつ明白な生命の危機が生じている状況での研究に関する要件の全てを満たしていることを確認するための手順 39](#_Toc197695287)

[２４．研究対象者等に経済的負担又は謝礼があればその内容 39](#_Toc197695288)

[２５．疾病等及び不具合が発生した場合の対応 39](#_Toc197695289)

[２６．健康被害に対する補償の有無及びその内容 40](#_Toc197695290)

[２７．不適合報告 41](#_Toc197695291)

[２８．研究の終了、中止 41](#_Toc197695292)

[２９．研究対象者の健康、遺伝的特徴に関する重要な知見が得られる可能性がある場合の研究結果（偶発的所見を含む）の取扱い(研究結果の開示の方針、開示の方法等) 42](#_Toc197695293)

[３０．研究に関する業務の一部を委託する場合には、当該業務内容及び委託先の監督方法 42](#_Toc197695294)

[３１．本研究で得られた試料・情報を将来の研究に用いる可能性 42](#_Toc197695295)

[３２．モニタリング及び監査の実施体制及び実施手順 42](#_Toc197695296)

[３３．知的財産権、所有権の帰属先 43](#_Toc197695297)

[３４．参考資料・文献リスト 43](#_Toc197695298)

# １．研究の名称

　　妊孕性温存を希望する子宮頸癌IB2-IB3期に対する主治療前化学療法を用いた縮小手術（CC-NAC C: FepCC）

# ２．研究の実施体制（実施医療機関の名称及び研究者等の氏名を含む）

　　本研究は以下の体制で実施する。

【統括管理者・研究責任医師】

　　所属：岡山大学学術研究院医歯薬学域　周産期医療学講座　職名：医師　氏名：長尾 昌二

住所：〒700-8558　岡山県岡山市北区鹿田町2-5-1

電話番号：086-235-7320（医局直通）

【モニタリング担当責任者】

　　所属：岡山県南西部（笠岡）総合診療医学講座　職名：医師　氏名：光井　崇

　　電話番号：086-235-7320（平日：8時～18時）（医局直通）

# ３．研究の背景、目的、意義

【研究の背景】

（１）子宮頸癌の疫学

　日本国内で年間約10000人が子宮頸癌に罹患し、3000人が死亡していると推測される^1)^。子宮頸部へのヒトパピローマウイルス（HPV）の持続感染を原因とする本疾患は若年女性に好発する。特に30歳代では乳癌に次ぐ第２位の罹患数であり、子宮頸癌の42％は45歳未満の妊娠可能年齢に発症する。

一方、日本人女性の平均初産年齢は昭和50年(1975年)時点では25.7歳であったのに対し、令和３年(2021年)時点では30.9歳と上昇傾向にある^2)^。子宮摘出術は初期および局所進行子宮頸癌の治療の主要部分を成すが、本術式は妊娠可能年齢の女性が妊孕能を喪失する要因の一つであり、妊娠・分娩に多大な影響を及ぼしうる。HPVワクチンの普及により将来的には減少が期待されているものの、本邦ではいまだその効果は確認されておらず、若年の初期あるいは局所進行子宮頸癌患者の妊孕性温存の需要は大きい。

（２）子宮頸癌に対する妊孕性温存手術の現状

　子宮頸がん治療ガイドライン2022年版によると、妊孕性温存手術を希望する妊娠可能年齢の子宮頸がん患者に対し、ⅠA1期には子宮頸部円錐切除術、ⅠA2期およびⅠB1期には広汎性子宮頸部摘出術による子宮温存が推奨されている^3)^。しかし、たとえ腫瘍が子宮頸部に限局していたとしても、腫瘍径2cmを超えるⅠB2期、ⅠB3期には広汎性子宮全摘術が推奨されており、妊孕性温存手術は適用されない。一方、広汎性子宮頸部摘出術は、腫瘍径2cm以下の病巣が子宮頸部に限局した子宮頸癌においては腫瘍学的な安全性が確認されているものの、流早産や出血などのトラブルが多く、周産期学的予後には限界がある^4-6)^。

（３） 諸外国の取り組み

　SHAPE試験およびConCerv試験の結果から腫瘍径2cm以下の初期子宮頸癌に対し、骨盤リンパ節郭清術を伴った単純子宮全摘術あるいは子宮頸部円錐切除術を行うことで広汎性子宮全摘術と同等の予後が得られることが明らかになった^7)8)^。これらの試験結果により、腫瘍径2cm以下（IB1期以下）の子宮頸癌に腹腔鏡下単純子宮全摘術や子宮頸部円錐切除術などの縮小手術の適用が可能であることが世界的なコンセンサスとなっている。また、LACC試験やSUCCOR cone試験でも腫瘍径2cm以下の症例において子宮頸部円錐切除術で断端に腫瘍の露出を認めなかった場合には、腹腔鏡下単純子宮全摘術により極めて良好な予後が得られることが示された^9)10)^。このように、腫瘍径2cm以下（IB1期以下）であることは、子宮頸癌に対する縮小手術を適用するにあたっての重要な基準と考えられる。

　現在、欧米諸国において径2cmを超える腫瘍（IB2期以上）を有する若年子宮頸癌患者の妊孕性温存に主治療前化学療法（Neoadjuvant chemotherapy：NAC）によって対応しようという動きが進んでいる^11)^。Maneoらは腫瘍径3cm以下、40歳以下のIb1期51例にNAC（cisplatin 75mg/m^2^ + paclitaxel 175mg/m^2^ + Ifosfamide 5g/m^2^）3サイクル後に腹腔鏡下骨盤リンパ節郭清術および子宮頸部円錐切除術、迅速病理検査で活動性の残存腫瘍があった場合にのみ広汎性子宮全摘術を施行した。21/51例（41%）において活動性の残存腫瘍を認めず、子宮頸部円錐切除のみで手術を終了した。これら21例では再発はなく、妊娠を目指した9例中6例が妊娠した^12)^。

MarchioleらはIB～IIA1期（腫瘍径30～45mm）の7例に対し、NAC（cisplatin 75mg/m^2^ + paclitaxel 175mg/m^2^ + Ifosfamide 5g/m^2^）3サイクル後に腹腔鏡下骨盤リンパ節郭清術および腟式広汎性子宮頸部切除術を施行した。1例に子宮傍組織浸潤を認め、腔内照射を追加したが、22ヶ月のfollow up期間で再発は認めていない^13)^。以上のように、NACにより腫瘍が大きく縮小した症例に限定すれば、子宮頸部円錐切除術を含む縮小手術を適用できることが推測される。

しかし、NACにより腫瘍径が2cm以下に縮小した子宮頸癌を対象とした縮小手術に関しての多くの症例報告やケースシリーズがあるものの、まとまった研究はない。

（４）妊孕性温存を希望する子宮頸癌患者に対するNAC後の子宮頸部円錐切除術を検討する意義

　腫瘍径が2cmを超える子宮頸癌に対して、NAC後に子宮頸部円錐切除術を行い、腫瘍学的安全性が確認できれば、妊孕性温存の大幅な適用拡大と同時に周産期的トラブルの回避が可能になる。このことは、妊孕性温存を希望する局所進行子宮頸癌患者に多大な恩恵をもたらすことが期待される。

【研究の目的】

　本研究の目的は、腫瘍径２cmを超える局所進行子宮頸癌（FIGO進行期IB2〜IB3期（2018年分類）の扁平上皮癌、腺癌、腺扁平上皮癌）患者を対象に、NAC後に子宮頸部円錐切除術および腹腔鏡下骨盤リンパ節郭清術を行い、その腫瘍学的安全性及び周産期医学的・女性医学的妥当性を確認することである。本研究によりその安全性、有用性が確認されれば、妊孕性温存を希望する若年の子宮頸癌IB2-IB3期の女性に新たな治療選択肢を提供できる可能性がある。

# ４．研究の方法

# （１）研究の種類・デザイン

　単施設・前向き介入研究

　非盲検、単群、第II相試験

　（２）研究のアウトライン（研究のフローチャート参照）

　文書による同意を取得し、すべての選択基準を満たし、除外基準のいずれにも合致しない患者を適格例とする。研究対象者から文書同意を取得し、登録前検査（スクリーニング検査）の後、適格性を最終的に確認し、登録を行う。登録患者に対しdose dense TC療法を実施する。

　dose dense TC療法3サイクルを実施可能であった症例のうち、骨盤MRI（内診、コルポスコープ検査、経腟超音波検査でも確認）で腫瘍径≤2cmかつ新規病変がないこと、PET-CTまたは骨盤〜頸部CT検査にて新規病変の出現がないことを確認できた患者に対し、子宮頸部円錐切除術を実施する。脱落患者には、開腹下に広汎性子宮全摘術を行う。

　　子宮頸部円錐切除術の摘出物病理組織検査で、病理学的に腫瘍径≤2cmかつ断端陰性が確認できた患者に対し、腹腔鏡下骨盤リンパ節郭清術を実施する。リンパ節転移を認めた場合には、シスプラチン隔週投与を併用した同時化学放射線療法を実施する。

　　脱落患者も含め全ての登録患者に対し2年間の経過観察を行う。

　主要評価項目を子宮温存可能であった患者の割合、副次評価項目を2年無再発生存割合、2年全生存割合、無再発生存期間、全生存期間、女性医学的予後（QOLを含む）、月経再開に至った患者の割合・排卵再開に至った患者の割合、 妊娠に至った患者の割合、分娩に至った患者の割合、毒性の種類、グレードおよび発生割合（CTCAE ver.5.0を用いる）とする。

　　５例が登録された時点で子宮温存可能であった患者が2例以下の場合には試験の継続の妥当性について検討する。

（３）研究対象者の研究参加予定期間（研究対象者単位での介入期間）

　130週間（約2年半）

　スクリーニング期：4週間

　介入期間：22週間（NAC: 12週間、子宮頸部円錐切除術: 5週間、腹腔鏡下骨盤リンパ節郭清術: 5週間）

　後観察期間：104週間(2年間)

（４）介入する医薬品・医療機器の概要とその介入内容

①　概要

| 一般名 | パクリタキセル |
| --- | --- |
| 商品名／製造販売業者 | パクリタキセル注 100 mg/16.7mL（日本化薬株式会社） |
| 剤形・性状・含量 | 無色〜微黄色澄明の粘稠性の液。pH3.0〜7.0(10v/v%水溶液) |
| 薬効分類 | 抗悪性腫瘍剤 |
| 効能・効果 | 進行または再発の子宮頸癌 |
| 用法・用量 | 通常成人にはパクリタキセルとして1日1回135mg/㎡（体表面積）を24時間かけて点滴静注し、少なくとも３週間休薬する。これを１クールとして投与を繰り返す。 |
| 保管条件 | 室温保存  包装開封後もバイアルを箱に入れて保存すること。 |

| 一般名 | カルボプラチン |
| --- | --- |
| 商品名／製造販売業者 | カルボプラチン点滴静注液 150 mg/15mL（ヴィアトリス・ヘルスケア合同会社、日本化薬株式会社） |
| 剤形・性状・含量 | 無色澄明〜微黄色澄明の液。pH5.5〜6.5 |
| 薬効分類 | 抗悪性腫瘍剤 |
| 効能・効果 | 子宮頸癌 |
| 用法・用量 | 通常、成人にはカルボプラチンとして、1日1回300〜400mg/㎡（体表面積）を投与し少なくとも4週間休薬する。これを１クールとし、投与を繰り返す。なお、投与量は、年齢、疾患、症状により適宜増減する。 |
| 保管条件 | 本剤は、光及び熱により分解するので、直射日光や高温を避け、外箱開封後は遮光して保存すること。  冷蔵庫保存では、結晶が析出することがある。 |

②　介入内容

本研究は3段階の介入を行う。まず、適格症例に対して介入１を行い、要件を満たした症例に対してのみ介入2、さらに要件を満たした症例に介入3を行う。

【介入１：NAC】

　NACとしてdose dense TC療法は、paclitaxel 80mg/m^2^ (day1, 8, 15静脈内点滴投与) +carboplatin AUC =6 (day1静脈内点滴投与)を1サイクルとして 21日ごとに実施する。

1. 体表面積の計算

　薬剤の投与量の計算に用いる体表面積の算出にはDuBoisの計算式を用いる。

　DuBoisの計算式：BSA= Body Weight^0.425^×Height^0.725^×71.84/10,000

　BSA （m^2^）, Body Weight （kg）, Height （cm）

　薬剤の投与量の計算の際、体表面積の最大値は2.0m^2^とする。

2. Paclitaxelの投与量の計算

　DuBoisの計算式より算出した体表面積より計算する。

3. Carboplatinの投与量の計算

　Carboplatinの投与量は下記に示すCalvertの計算式を用いる^14)^。

　Calvert の計算式：Carboplatin投与量（mg/body）＝AUC目標値×（GFR＋25）

1) 本試験ではGFRはクレアチニン・クリアランス（Ccr）と同等とみなす。

2) Ccrは下記のCockcroft-Gaultを使用して算出する^15)^｡

Cockcroft-Gaultの計算式：CCr =0.85× ｛(140-age) ×BW｝/(72×serum creatinine × 1.73）

Ccr （ml/min）, age （years）, BW （kg）, serum creatinine （mg/dl）

3) 血清クレアチニン値が0.6mg/dL未満の症例では血清クレアチニン値を0.6 mg/dLに補正して計算する。

4) 新たな尿路閉塞やCTCAE Grade2以上の腎機能障害（血清クレアチニン値が施設の正常上限値の1.5倍を超える）がない場合には次サイクル以降は再計算しなくてもよい。

5) Carboplatinの最大投与量は1000mg/bodyとする。

4. Paclitaxelの投与方法

1) 前投薬実施の後、規定の投与量を250mlの5％ブドウ糖液または生理食塩水に溶解し、専用ラインを用いて1時間で点滴静注する。

2) 各サイクルのDay1, 8, 15に投与する。

5. Carboplatinの投与方法

1) Paclitaxelの投与に引き続き、規定の投与量を250mlの5％ブドウ糖液または生理食塩水に溶解し1時間で点滴静注する。

2) 各サイクルのDay1に投与する。

6. 投与開始基準

1) 1サイクル目Day1開始基準

　投与開始2週間前までに患者選択基準に記載された臨床検査値等すべてを満たすことを確認する。

2) 1サイクル目Day8, Day15開始基準

　投与開始前2日以内に下記の基準をすべて満たしていることを確認する。開始基準をすべて満たしていない場合には投与を最大で3週間まで延期することができる。3週間延期をしても開始基準を満たさない場合にはdose denseTC療法を中止する。

好中球数 500/mm^3^以上

血小板数 50,000/mm^3^以上

3) 2サイクル目以降Day1開始基準

　投与開始前2日以内に下記の基準をすべて満たしていることを確認する。開始基準をすべて満たさない場合には投与を最大で3週間まで延期することができる。3週間延期をしても開始基準を満たさない場合にはdose denseTC療法を中止する。

好中球数 1,000/mm^3^以上

血小板数 75,000/mm^3^以上

非血液毒性 Grade1以下

（脱毛、疲労、悪心、便秘を除く）

4) 2サイクル目以降Day8, Day15開始基準

　投与開始前2日以内に下記の基準をすべて満たしていることを確認する。開始基準をすべて満たしていない場合には投与を最大で3週間まで延期することができる。3週間延期をしても開始基準を満たさない場合にはdose denseTC療法を中止する。

好中球数 500/mm^3^以上

血小板数 50,000/mm^3^以上

7. 投与量減量の手順

　減量基準に1つでも該当した場合は次サイクル以降の投与量を薬剤Levelに従い1Level減量する。減量基準に複数該当した場合にも次サイクルの減量は1 Levelずつ実施する。（一度に2 Levelは減量しない）減量後に再度減量基準に抵触した場合には次サイクルにおいても薬剤Levelに従いさらに1 Level減量する。減量回数は2回までとし、3回以上の減量は行わない。2回の減量後に再度減量基準に抵触した場合には次サイクル以降のdose denseTC療法を中止する。一度投与量を減量した場合には再増量はそれ以後行わないものとする。

投与量減量基準

① PaclitaxelおよびCarboplatinの2剤を1Level減量する場合

・投与開始基準を満たさず、2週間を超えて（15日以上）3週間まで（21日以下）の延期を要した場合

・前サイクル中に好中球減少（DLT−ANC）^*1^もしくは血小板減少（DLT−PLT）^*2^をきたした場合

・前サイクル中にGrade3の非血液毒性（脱毛、疲労、悪心、便秘、末梢神経障害、体重増減を除く）を認めた場合

② Paclitaxelのみ1Level減量する場合

・前サイクル中にGrade2以上の末梢神経毒性を認めた場合

^*1^ 好中球減少（DLT−ANC）とは下記①②のように定義する。

① 発熱性好中球減少

② G-CSF製剤の使用にもかかわらず7日以上持続するGrade4の好中球減少（好中球数500/mm^3^未満）

^*2^ 血小板減少（DLT−PLT）とは下記①②のように定義する。

① Grade4 （<25,000/mm^3^未満）の血小板減少。

② 出血傾向を伴う血小板減少（25,000以上50,000/mm^3^未満）を認めるか、血小板輸血を必要とした場合。

毒性による投与量の減量

　　Level 　　Paclitaxel (mg/m^2^) Carboplatin (AUC)

　　 0 80 6

　　 -1 70 5

　　 -2 60 4

　　 -3 中止 中止

8. Paclitaxelに対する過敏反応の対応

　Paclitaxelに対する過敏反応は用量制限毒性とはしない。過敏反応に対する治療後に全量投与することも可能である。過敏反応後に再投与する際には投与速度を遅らせることが望ましい。

9. 投与量の再計算を要する場合

1) 腎機能障害が発現した場合の対応

　血清クレアチニン値が施設基準の上限値の1.5倍を超えている場合はcarboplatin投与量を各投与サイクルにおいて再計算する。

2) 体重変動による投与量の補正

　体重の増減がdose denseTC療法開始時の体重と比較して10％未満の場合、次サイクルの投与量の再計算による補正は必須としない。10％以上の体重の増減があった場合には投与量の再計算を行う。

投与量の再計算時の体重から、さらに±10％以上の増減があった場合は、再度、投与量を計算する。

10. dose denseTC療法終了・中止基準

1) dose denseTC療法の終了基準

　 dose denseTC療法は原則として3サイクルをもって終了とする。

2) dose denseTC療法の中止基準

① 有害事象によりdose denseTC療法の継続ができない場合。

　・3週間を超える遅延が発生した場合*^1^。

　・Grade4の非血液毒性が発生した場合。

　・Level-3の減量が発生した場合*^1^。

② 患者がdose denseTC療法の中止を申し出た場合。

③ dose denseTC療法中の死亡の場合。

④ dose denseTC療法開始後に原病の増悪・再発が認められた場合。

⑤ その他の理由で本試験の継続が好ましくないと試験担当医が判断した場合。

11. 併用・支持療法

1) G-CSF

① G-CSFの予防投与は行わない。

② 原則としてASCOガイドラインに沿って以下の場合にG-CSFに使用を考慮して良い。

　・投与開始条件

　好中球数1,000/mm^3^（Grade3）未満で発熱（38℃以上）が認められた場合。

　好中球数500/mm^3^（Grade4）未満の場合。

　前サイクルで上記事象が観察され、好中球数1,000/mm^3^未満が観察された場合。

　・使用量および使用方法

　50μg/m^2^1日1回皮下注射

　・再開時期

　好中球数が最低値を示した後5000/mm^3^以上に達した場合。

　好中球数が2,000/mm^3^以上に回復し感染症が疑われるような症状がなく、本剤に対する反応性　から患者の安全が十分に確保できると判断した場合。

2) 貧血に対する治療

　貧血の治療上必要な場合は、鉄剤、輸血を行う。

3) 制吐剤

　悪心・嘔吐の軽減を目的とした5-HT3 antagonistおよび他の制吐剤の予防的投与が推奨される。

4) 抗アレルギー剤

　Paclitaxelによるアレルギー反応の予防を目的としたステロイドおよび抗ヒスタミン薬など予防的投与が推奨される。

5) Premedication

① Paclitaxel投与30分前にジフェンヒドラミン50mg 経口投与、デキサメサゾン20mg 静脈内投　与、塩酸ラニチジン50mg 静脈内投与のShort Premedicationを採用する。

② 1サイクル目でアレルギー症状がなかった場合には次サイクル以降デキサメサゾンの投与量を８mgから適宜漸減してもよい。

6) 許容されるその他の併用・支持療法、手術

① 合併症および有害事象の治療を目的とした薬剤（抗菌剤、鎮痛剤、輸血など）の併用が必要と判断された場合は適宜用いることができる。

② Paclitaxelによる末梢神経障害対策を目的とする各種対症療法は許容される。

7) 許容されない併用・支持療法、手術

① 悪性腫瘍の治療を目的とした薬剤の投与を含む一切の治療はdose dense TC療法の終了・中止まで許されない。これらにはPaclitaxelおよびCarboplatin以外のすべての抗がん剤、免疫療法、放射線治療などが含まれる。

② 他の治験薬の投与も許容されない。

12. 評価方法

　１サイクル施行ごとに内診、コルポスコープ検査、経腟超音波検査、骨盤MRIにて腫瘍サイズを確認する。

【介入２：子宮頸部円錐切除術】

　実施に必要な要件は以下の通りとする。

① コルポスコピーおよび経腟超音波検査、骨盤MRI検査で最大腫瘍径2cm以下である。

② 骨盤MRI検査で正常な間質が3mm以上残存し間質浸潤が50％未満、かつ新規病変がない。

・子宮頸部円錐切除術は全身麻酔、脊椎麻酔を問わない。

・3時9時の子宮動脈下行枝を結紮したのち腫瘍から5mm以上離して全周性に絹糸でマーキングを行い牽引しながら切除する。

・頸部腫瘍を完全に取り除くことができる範囲の切除を行うが、膀胱子宮窩腹膜やダグラス窩腹膜は開放しない。

・腫瘍が認識できない場合（NACが完全奏効した場合）には、2cmの奥行きで十分な広さの子宮頸部を切除する。

術後病理診断にて、明らかに最大腫瘍径2cm以下で切除断端陽性あるいは焼灼による評価不能の場合には再度子宮頸部円錐切除術を実施することを検討する。2回子宮頸部円錐切除術を実施した場合には、腫瘍径や間質浸潤の深さをそれぞれ合計し、リスク評価する。

尚、病理学的腫瘍径2cm以下、かつ間質浸潤10mm以下を満たさない場合には開腹広汎性子宮全摘術を実施する。新規病変を認めた場合には、放射線治療（同時化学放射線療法を含む）あるいは化学療法を考慮する。

【介入3：腹腔鏡下骨盤リンパ節郭清術】

　実施に必要な項目は以下の通りとする。

切除した組織について病理学的に、

① 腫瘍径2cm以下であること。

② 間質浸潤10mm以下であること。

③ 切除断端陰性であること。

④ 脈管侵襲陰性であること。

要件を満たす症例に対して腹腔鏡下骨盤内リンパ節郭清術を行う。

・日本産科婦人科内視鏡学会技術認定医（腹腔鏡）と日本婦人科腫瘍学会婦人科腫瘍専門医の協力体制の下で、あるいは腹腔鏡手術手技に十分習熟した日本婦人科腫瘍学会婦人科腫瘍専門医が執刀する。

・使用デバイス、ポート配置や郭清手技の手順は規定しない。

・リンパ節の郭清範囲は所属リンパ節とし、両側の全てのリンパ組織を摘除する。所属リンパ節の名称と位置は子宮頸癌取り扱い規約に従い、以下のものとする。すなわち、総腸骨リンパ節、外腸骨リンパ節、内腸骨リンパ節、閉鎖リンパ節、仙骨リンパ節、基靭帯リンパ節、鼠経上リンパ節である。

・手術の標準化及び適切な郭清範囲の担保を目的として、所属リンパ節摘出個数は 20 個（病理学的個数）を目安とする。

・骨盤リンパ節郭清術後、肛門挙筋、内閉鎖筋、尾骨筋が同定できる膀胱側腔、下腹神経と内腸骨静脈が同定できる直腸側腔を左右それぞれ静止画で保存し、手術の質の担保の指標とする。

　尚、以下のいずれかに当てはまる場合には介入3を実施しない。

① 術中、播種病変や卵巣への転移を認めた場合には直ちに手術を中止する。

② 腫大リンパ節を発見した場合には迅速病理検査に提出し、転移の有無を確認する。転移であれば、開腹下に広汎性子宮全摘術を行う、または、複数のリンパ節転移を認める場合には手術を中止する。その後の治療方針は日常診療に従う。

③ 術後病理診断で新たにリンパ節転移を認めた場合には、放射線治療（同時化学放射線療法を含む）を実施する。

## （５）被験薬の管理方法

　　本研究で用いる薬剤は既承認であり、 医薬品等の承認事項に基づく適切な保管等の管理を行う。

（６）併用薬（療法）等に関する規定

１）併用薬（療法）：通常の臨床に準ずる。化学療法に伴う毒性の低減は予防的対応も含めて積極的に実施する。

２）併用禁止薬（療法）：放射線療法、抗がん剤、免疫療法剤、ホルモン剤を含む全ての抗がん治療の併用を禁止する。

## （７）症例登録

　研究責任医師あるいは研究分担医師は、以下の手順で症例登録を行う。

１）すべての選択基準を満たし、除外基準のいずれにも合致しない患者を適格例とし、文書による同意を取得する。

２）研究責任医師が保管する研究対象者識別コードリスト（対応表）を作成し、同意取得日及び研究対象者と研究対象者識別コードを対応させるために必要な事項を記載する。

３）同意撤回、中止、脱落等が生じた時は、速やかに研究責任医師に報告する。

（８）研究終了後の対応

本研究終了後は、この研究で得られた成果も含めて、研究責任医師は研究対象者に対し最も適切と考える医療を提供する。

# ５．観察及び検査項目

## （１）研究対象者背景

　年齢、合併症、既往歴

## （２）検査項目

＜スクリーニング期＞

　患者背景情報

身体所見：PS(Performance Status)、身長、体重

診察所見：PS、内診所見、直腸診所見、コルポスコピー所見、経腟超音波で測定した腫瘍径

造影MRI（アレルギー歴のある場合は単純でも可）：腫瘍径の確認

PET-CT（造影CTでも可）：遠隔転移の有無

FACT-CX、FSFI、HADS質問表：女性医学的評価

＜介入期間＞

身体所見：PS、体重

診察所見：内診所見、コルポスコピー所見、経腟超音波での腫瘍径（NAC中2、3サイクル目開始前、子宮頸部円錐切除術前、腹腔鏡下リンパ節郭清術前）

造影MRI：残存腫瘍径の確認（NAC中2、3サイクル目開始前、子宮頸部円錐切除術前、腹腔鏡下リンパ節郭清術前）

PET-CT（造影CTでも可）：遠隔転移の有無（子宮頸部円錐切除術前）

FACT-CX、FSFI、HADS質問表：女性医学的評価（腹腔鏡下リンパ節郭清術後）

子宮頸部円錐切除術後組織：病理学的腫瘍径、切除断端の腫瘍組織の有無、間質浸潤の深さ

切除リンパ節：組織学的リンパ節転移の有無（腹腔鏡下リンパ節郭清術後）

＜後観察期間＞

腟部細胞診：再発の有無（3ヶ月毎）

造影CT：再発の有無（6ヶ月毎）

FACT-CX、FSFI、HADS質問表：女性医学的評価

月経の有無、排卵の有無（基礎体温表）

## （３）自他覚症状の確認

　問診により確認する（全ての期間を通して行う）。

## （４）有害事象と副作用の確認（スクリーニング時、dose dense TC療法中、腹腔鏡下リンパ節郭清1ヶ月後まで）

　有害事象の内容、発現時期・消失時期、程度、処置、転帰、重篤性評価等を症例報告書（case

report form：CRF）に記載する。必要があれば追跡調査を行う。

スケジュール表

| 項目 | 前観察期間 | 介入  開始日 | 介入期間 | | | | | 後観察期間 |
| --- | --- | --- | --- | --- | --- | --- | --- | --- |
| 時期 | 2～4週前 | 0週 | ddTC^＊１^  1サイクル後 | ddTC  2サイクル後 | ddTC  3サイクル後 | 子宮頸部円錐切除術後 | 腹腔鏡下リンパ節郭清術後 | 終了(中止) |
| 同意取得 | ● |  |  |  |  |  |  |  |
| 患者背景情報の確認 | ● |  |  |  |  |  |  |  |
| 介入期間 |  |  |  |  |  |  |  |  |
| 身体所見 | ● |  | ● | ● | ● |  |  |  |
| 診察 | ● |  | ● | ● | ● |  |  |  |
| 造影MRI | ●^＊2^ |  | ● | ● | ● |  |  |  |
| PET-CT | ●^＊2^ |  |  |  | ●^＊3^ |  |  |  |
| 病理組織検査 |  |  |  |  |  | ● |  |  |
| 病理組織検査 |  |  |  |  |  |  | ● |  |
| 造影CT | ●^＊2^ |  |  |  | ●^＊3^ |  |  | ● |
| 腟部細胞診 |  |  |  |  |  |  |  | ● |
| 質問表 | ● | ● | ● |  |  |  | ● | ● |
| 月経、排卵の有無 |  |  |  |  |  |  |  | ● |

＊1 dose dense TC療法

＊2 スクリーニング検査のうち造影MRI、PET-CT（または造影CT）については同意前１ヶ月以内の情報を用いてもよい。

＊3 ddTC３サイクル後の全身検索を目的とした画像検査はPET-CT・造影CT検査を問わない。

# ６．評価項目

## （１）主要評価項目（Primary endpoint）

子宮温存可能割合（全症例を対象とするが、CRTを実施した場合は子宮温存に含めない）

　設定根拠：臨床的に重要な指標であるため。

## （２）副次評価項目（Secondary endpoint）

　①　2年無再発生存割合(介入３の後、骨盤リンパ節転移陰性の症例)

　設定根拠：腫瘍学的安全性を評価するために重要かつ現実的な指標であるため。

②2年無再発生存割合（全症例を対象としたもの）

　設定根拠：腫瘍学的安全性を評価するために重要な指標であるため。

③2年全生存割合（全症例を対象としたものと介入３の後、骨盤リンパ節転移陰性の症例）

　設定根拠：腫瘍学的安全性を評価するために重要な指標であるため。

④無再発生存期間（全症例を対象としたものと介入３の後、骨盤リンパ節転移陰性の症例）

　設定根拠：腫瘍学的安全性を評価するために重要な指標であるため。

⑤全生存期間（全症例を対象としたものと介入３の後、骨盤リンパ節転移陰性の症例）

　設定根拠：腫瘍学的安全性を評価するために重要な指標であるため。

⑥女性医学的予後（QOLを含む）(介入３の後、骨盤リンパ節転移陰性の症例に限る)

　設定根拠：臨床的に重要な指標であるため。

⑦月経再開に至った割合・排卵再開に至った割合(介入３の後、骨盤リンパ節転移陰性の症例に限る)

　設定根拠：女性医学的・生殖内分泌医学的な有用性・安全性の評価に重要な指標であるため。

⑧妊娠に至った割合(介入３の後、骨盤リンパ節転移陰性の症例)

　設定根拠：生殖内分泌医学的な有用性・安全性の評価に重要な指標であるため。

⑨生児獲得割合、流産・早産割合(介入３の後、骨盤リンパ節転移陰性の症例)

　設定根拠：周産期学的な有用性・安全性の評価に重要な指標であるため。

⑩毒性の種類、グレードおよび発生割合（CTCAE ver.5.0を用いる）（全症例を対象とする）

　設定根拠：臨床的に有用性・安全性の評価に重要な指標であるため。

# ７．目標症例数及び設定根拠

　　当院では、被験治療(腫瘍径２cmを超える局所子宮頸癌に対するNAC後の子宮頸部円錐切除術および腹腔鏡下骨盤リンパ節郭清術による妊孕性温存療法)実施例として10例を予定している。

設定根拠：40歳未満の子宮頸癌IB2-3期患者数は、全国で年間約240人程度である。岡山県近隣を含む医療圏人口を3-400万人とし、人口比から年間6-8人程度の対象患者がいると推測されるため。

本試験では以下の仮説と統計的設定を用いる。

・期待値（目標成功率）：70%
・閾値（臨床的に価値があると判断される最低成功率）：45%
・有意水準：0.10（片側）
・検出力（Power）：80%

この設計で、10例中7例以上が子宮温存に成功した場合、p値が0.10未満となり、この治療法が有望であると統計的に評価できる。

後述のように、実際の研究では可能な限り日本全国から患者を募集する。

# ８．データの集計及び統計解析方法

（１）解析対象集団

解析対象集団はFull Analysis Set（FAS）とする。ここでFASを、同意を取得した症例のうち、被験治療を開始された全症例と定義する。

（２）解析方法

1. 解析対象集団

　　最大の解析対象集団（FAS; Full Analysis Set）を全ての登録された症例から最小限の除外可能な症例を除いた集団として定義する。最小限の除外可能な症例とは、適格ではなかった人が誤って登録されてしまった症例や、試験に割り付けられた後一度もdose dense TC療法が実施されなかった症例、割り付けの後のデータが全く無い症例とする。有効性に関する主解析はFASで行う。被験治療を開始されたすべての症例をSP（Safety Population）とし、安全性評価に関する解析対象集団とする。

2. 解析方法

　　統括管理者は、データ及びその他の必要なすべての報告書について、正確性、完全性及び適時性を保証する。原資料（患者の医療記録など）に由来する症例報告書で報告されたデータは、その原資料と一致するものでなければならない。

　　研究の途中において、同意撤回により脱落した患者に関するデータは利用しない。その他の理由により脱落した患者のデータは、脱落前までの利用可能なデータを利用する。

　　得られたデータは外部と接続されていないパソコン内に入力し、集計、解析を行う。データベースとして固定された変数について記述統計量を算出する。連続変数では、平均値、標準偏差、中央値、四分位範囲を示す。二値変数を含むカテゴリ変数では、頻度および割合を示す。

（３）欠損値、不採用および異常データの取り扱い

　　欠損値は可能な限り元資料を確認し、補充するように努める。それにも関わらず欠損値がある症例については除外する。また、入力間違いや測定機器のエラーを疑うような異常データについても元資料を確認し、適切な値であったかどうかを確認する。確認によっても値が修正されなかった場合は、元値で解析を行う。同意撤回、中止・脱落症例において、原則として同意撤回、中止・脱落までの情報等は研究に使用する。

（４）統計解析計画の変更手順

　　当初の統計解析計画を変更する場合は、統括管理者、研究責任医師および研究分担医師が協議した上で統計解析計画の変更を行い、倫理審査委員会の承認を受ける。

（５）中間解析

　　5症例に達した時点で中間解析を行う。子宮温存が可能であった症例が2例以下の場合には、患者登録を休止し、その後に試験の継続の可否を検討する。

（６）主要評価項目について

　　子宮温存可能割合

全症例を対象とするが、CRTを実施した場合は子宮温存に含めない。

　　介入1~3に該当しない場合や再発・死亡のイベント発生がした時点で子宮温存可能として扱わない。

# ９．研究実施期間

　　認定臨床研究審査委員会承認後のjRCT^＊^公表日から2030年3月31日

　（登録締切：2027年12月31日）

　　3年間の登録期間および2年間の経過観察期間を設定する。

＊jRCT：臨床研究等提出・公開システム（Japan Registry of Clinical Trials）

<https://jrct.mhlw.go.jp/>

# １０．研究対象者の設定方針

（１）選択基準

　　以下の基準を全て満たすものを対象とする。

　① 臨床的に子宮頸癌ⅠB2あるいはⅠB3期と診断された患者（FIGO2018年分類）（診断は内診、腟鏡診、直腸診、骨盤MRI、CTまたはPET-CT、経腟超音波検査などを用いて総合的に行う）

　② 病理組織学的に診断の確定した扁平上皮癌、腺癌、腺扁平上皮癌の患者

　③ 未閉経の患者

　④ 同意取得時点で40歳未満の患者

　⑤ 十分な全身の臓器機能を有する患者（臨床検査は手術予定日前28日以内に行われたものとする

好中球数 1,500 /mm^3^以上

血小板数 100,000 /mm^3^以上

AST（GOT）､ALT（GPT） 100 IU/L以下

血清総ビリルビン 　 1.5 mg/dl未満

血清クレアチニン 　 1.5 mg/dl未満

心電図 正常範囲または無症状でかつ治療を必要としない程度の異常 （心疾患､重篤な不整脈のない症例）

末梢神経障害 Grade1以下　（CTCAE ver.5.0）

　⑥ 妊孕性温存を希望する患者

　⑦ 本研究の参加にあたり十分な説明を受けた後、十分な理解の上、患者本人の自由意思による文書による同意が得られた18歳以上の患者

## （２）除外基準

　　以下の基準のいずれかに該当するものは、本研究に組み入れないこととする。

1. 病理組織学的にHPV-independent癌の患者
2. 全ての活動性の重複癌患者（同時性重複がん及び無病期間が5年以内の異時性重複癌。ただし皮膚の基底細胞癌と扁平上皮癌、並びに局所治療により治癒と判断される上皮内癌もしくは粘膜内癌相当の病変は活動性の重複がんに含めない）
3. 重篤な合併症を有する患者（重篤な心疾患又は脳血管障害､コントロール困難な糖尿病又は高血圧症､肺線維症、間質性肺炎､出血､活動性の消化性潰瘍又､重篤な神経疾患など）
4. ポリオキシエチレンヒマシ油（クレモホールＥＬ^R^）含有製剤（シクロスポリンなど）および､硬化ヒマシ油含有製剤（注射用ビタミン剤など）の投与歴に関連して過敏症が発現したことのある患者
5. 治療を要する活動性の感染症患者
6. 妊娠､授乳中及び妊娠している可能性のある患者
7. 未成年者や自身の判断で同意することができない患者
8. その他、研究責任医師、研究分担医師が研究対象者として不適当と判断した患者

# １１．研究の背景及び科学的合理性の根拠（研究の合理性・妥当性）

　3. 研究の背景、目的、意義に示した通り、本研究の目的はIB2期、IB3期の子宮頸癌を対象に子宮頸部円錐切除術および腹腔鏡下骨盤リンパ節郭清術を行い、その腫瘍学的安全性および女性医学的、周産期医学的妥当性を検証することである。手術に先立ってNACを施行し、腫瘍径2cm以下に縮小した症例に限定して妊孕性温存の対象とする。本研究によりその安全性、有用性が確認されれば、妊孕性温存を希望する若年の局所進行子宮頸癌女性に妊孕性温存手術という新たな治療選択の提供が可能になる。

（１）局所進行子宮頸癌に対するNACの意義

　　長年にわたって局所進行子宮頸癌に対するNACを用いた予後改善を検証する臨床試験が試みられてきた。しかし、2つの大規模なランダム化試験で標準治療である同時化学放射線療法に対するNAC後の広汎性子宮全摘術の優位性は示されず、局所進行子宮頸癌に対する標準治療は同時化学放射線療法とされている^16)17)^。NACによる予後の改善は認められなかったものの、その高い奏効割合が縮小手術や術後の放射線治療回避につながる可能性については現在も議論が続いている。

　　Vercellinoらは、腫瘍径が2cmを超える子宮頸癌18例に対し腹腔鏡下骨盤及び傍大動脈リンパ節郭清術によるリスク分類を行い、リンパ転移を認めた症例には同時化学放射線療法、認めなかった症例にはNAC後に広汎性子宮頸部全摘術による子宮温存を試みた。その結果、前者(12例)では3/12例（25%）に再発を認めたのに対し、後者（6例）では再発を認めなかった^18)^。また、Kimらのメタアナリシスでは、ⅠB1〜ⅡA期（FIGO2009）の子宮頸癌に対してNACを行うことで、腫瘍径、リンパ節転移の縮小、遠隔転移の縮小が認められ、術後放射線療法の必要性を減らせる可能性が明らかになっている^19)^。

（２）腫瘍径2cm以下の子宮頸癌に対する縮小手術

　　2011年に腫瘍径2cm以下の子宮頸癌における子宮傍組織浸潤は１％未満と非常に稀であり、このような患者においては広汎性子宮全摘術が必要でない可能性が示された^20)^。それを前方視的に検証することを目的にSHAPE試験が計画された。この試験は、IA2期からIB1期（腫瘍径2cm下）の子宮頸癌を対象に、広汎性子宮全摘術と単純子宮全摘術（いずれの場合も骨盤リンパ節郭清術は実施）を比較した大規模なランダム化試験である。その結果、骨盤内再発、骨盤外再発ともに両者の間に有意差がないことが示された^7)^。

さらに、ConCerv試験では、IA2期からIB1期（腫瘍径2cm以下）の子宮頸癌を対象に、子宮頸部円錐切除術後の骨盤リンパ節生検（42例）、子宮頸部円錐切除術後の単純子宮全摘および骨盤リンパ節生検（36例）、単純子宮全摘後の骨盤リンパ節郭清術（16例）の2年再発率が比較され、それぞれ2.4%、0%、12.5%であり、早期子宮頸癌患者において、子宮頸部円錐切除術および骨盤リンパ節生検による治療の可能性が示された^8）^。

SEERのデータ解析によると、ⅠB1期（FIGO2008年分類）2571例において子宮頸部円錐切除術、子宮頸部切除術、単純子宮全摘術と広汎性子宮全摘術及び準広汎性子宮全摘術の間に10年疾患特異生存率に差がなく、死亡率を上げる因子は、リンパ節転移陽性、2cmを超える腫瘍径であることが明らかにされた^21)^。

以上のように、リンパ節転移陰性、かつ、腫瘍径2cm以下の子宮頸癌に対する子宮頸部円錐切除術を含む縮小手術の妥当性が示されている。

（３）子宮頸癌に対する子宮頸部円錐切除術実施の要件

　　広汎性子宮全摘術および広汎性子宮頸部切除術と子宮頸部円錐切除との間での最も大きな差異は子宮傍組織の切除の有無である。すなわち本研究において重要なことは、傍組織への浸潤がない症例を確実に抽出することである。

　　Convensらの研究では、根治的子宮全摘術を受けたIA1期からIB1期の子宮頸癌患者842人の傍組織浸潤を評価した^22)^。 その結果、33例（4％）に傍組織浸潤が認められ、腫瘍の大きさ、脈管侵襲、浸潤の深さ、骨盤リンパ節転移陽性と関連していた。また腫瘍径２cm以下、骨盤リンパ節転移陰性、間質浸潤が10mm未満の症例について傍組織浸潤が見られたのは、わずか0.6%であった。また、Stegemanらの研究では、IA1期からIB1期の閉経前の子宮頸癌患者103人の傍組織浸潤を評価した^23)^。 全ての患者は腫瘍径2cm以下、間質浸潤10mm以下、骨盤リンパ節転移陰性であったが103例中2例（1.9％）だけが傍組織への浸潤を認めており、この2例はいずれも脈管侵襲を有していた。

　　これら研究の結果を踏まえ、腫瘍径２cm以下、間質浸潤10mm以下、脈管侵襲陰性、骨盤リンパ節転移陰性の全ての条件を満たす腫瘍については傍組織浸潤を認める可能性は極めて低いと考えられ、これらが子宮頸癌に対する子宮頸部円錐切除術適用の条件と考えられる。

（４）NACとしてdose dense TC療法を選択する妥当性

　　研究代表者等は、以前より局所進行子宮頸癌を対象にプラチナ製剤およびタキサン製剤を用いたNAC後の広汎性子宮全摘術の臨床試験を行い、良好な成績を上げてきた^24-27)^。dose dense TC療法は、22%の病理学的完全奏効を含む92%に奏効し、98%の患者で広汎性子宮全摘術の実施が可能であった^25)^。2年および3年無再発生存期間および全生存期間は、88.0%、83.8%および98%、95.4%であった。しかも、IB3期での再発は（IB2期は対象に含まず）、1/13例のみであった。

このように、本試験で用いるNACのレジメンとしてdose dense TC療法を選択することに妥当性があると考えられる。

（５）研究グループのNACの経験

　　本研究の開始準備状況として、局所進行子宮頸癌IB3〜IIB期（FIGO2018年分類）に対する「dose dense TC療法を用いた術前化学療法の前向きコホート研究-Aパート」(研2203-043)を当院の倫理委員会の承認を得た上で2022年3月より実施している。すでにdose dense TC療法によるNACの使用経験を30例以上積んでいる。また、術後病理標本を用いて縮小手術、縮小子宮温存手術の切除範囲内に残存病変が残存しているかどうかのマッピングと術前画像との一致率を検討する研究「dose dense TC療法を用いた術前化学療法の前向きコホート研究-Bパート」(研2203-050)を実施中である。10症例について病理組織学的評価を病理専門医とともに実施した。さらに2023年より腹腔鏡下後腹膜リンパ節郭清術を導入し、50例以上の経験を積んでいる。

　　以上のように本研究は十分な合理性・妥当性を有すると考えられる。

# １２．インフォームド・コンセントを受ける手続き

　　研究責任医師または研究分担医師は、認定臨床研究審査委員会の承認が得られた説明文書を研究対象者本人に渡し、文書及び口頭による十分な説明を行い、自由意思による同意を文書で得る。研究対象者の同意に影響を及ぼすと考えられる有効性や安全性等の情報が得られたときや、研究対象者の同意に影響を及ぼすような実施計画等の変更が行われるときは、速やかに研究対象者に情報提供し、研究等に参加するか否かについて研究対象者の意思を予め確認するとともに、事前に認定臨床研究審査委員会の承認を得て説明文書・同意文書等の改訂を行い、研究対象者の再同意を得る。

　　同意説明文書には、以下の内容を含むものとする。

① 実施する特定臨床研究の名称、当該特定臨床研究の実施について実施医療機関の管理者の承認を得ている旨及び厚生労働大臣に実施計画を提出している旨

② 統括管理者の氏名又は名称、研究責任医師の氏名及び職名並びに実施医療機関の名称

③ 特定臨床研究の対象者として選定された理由

④ 特定臨床研究の実施により予期される利益及び不利益

⑤ 特定臨床研究への参加を拒否することは任意である旨

⑥ 同意の撤回に関する事項

⑦ 特定臨床研究への参加を拒否すること又は同意を撤回することにより不利益な取扱いを受けない旨

⑧ 特定臨床研究に関する情報公開の方法

⑨ 特定臨床研究の対象者の求めに応じて、研究計画書その他の特定臨床研究の実施に関する資料を入手又は閲覧できる旨及びその入手又は閲覧の方法

⑩ 特定臨床研究の対象者の個人情報の保護に関する事項

⑪ 試料等の保管及び廃棄の方法

⑫ 特定臨床研究に対する医薬品等製造販売業者等の関与の有無とその内容

⑬ 苦情及び問合せへの対応に関する体制

⑭ 特定臨床研究の実施に係る費用に関する事項

⑮ 他の治療法の有無及び内容並びに他の治療法により予期される利益及び不利益との比較

⑯ 特定臨床研究の実施による健康被害に対する補償及び医療の提供に関する事項

⑰ 特定臨床研究の審査意見業務を行う認定臨床研究審査委員会における審査事項その他当該特定臨床研究に係る認定臨床研究審査委員会に関する事項

⑱ その他特定臨床研究の実施に関し必要な事項

　なお、本試験への参加にあたり、化学療法による卵巣機能への影響を考慮に入れる必要がある。そのため、未婚の参加者にはNAC開始前の卵子凍結、既婚の参加者にはNAC開始前の受精卵凍結保存の選択肢を提示する^17)^。また、治療の結果、子宮温存が不可能であった場合には、自己の子宮での妊娠は困難となることを説明する。

# １３．個人情報等の取扱い（個人情報の加工の方法を含む）

# 本研究に係わるすべての研究者は、「ヘルシンキ宣言」、「臨床研究法」並びに「個人情報保護法」を遵守して実施する。研究実施に係る試料・情報を取扱う際は、研究対象者に研究独自の研究用IDを割り振り、氏名と研究用IDとの対応表を作成する。元データからは氏名を削除し、研究対象者の秘密保護に十分配慮した上で研究に用いる。研究期間を通して対応表ファイルはパスワードをかけ、漏洩しないように厳重に保管する。研究の結果を公表する際は、氏名、生年月日などの直ちに研究対象者を特定できる情報を含まないようにする。また、研究の目的以外に、研究で得られた研究対象者の試料・情報を使用しない。

# １４．研究対象者に生じる負担、予測されるリスク及び利益、これらの総合的評価並びに当該負担及びリスクを最小化する対策

（１）予想される利益

本研究で実施する介入により実地臨床では妊孕性温存が適応とならない患者について、妊孕性を温存できる可能性がある。

（２）予想される不利益（副作用）

　　本研究は実地臨床では開腹下の広汎性子宮全摘術あるいはCRTが実施される患者を対象としている。

手術による切除範囲の縮小により腫瘍の遺残、あるいは再発のリスクが高まる可能性がある。

また、NACによる腫瘍縮小効果が得られなかった場合、NAC中の病勢悪化をきたす可能性がある。脱毛、末梢神経障害、卵巣機能障害をはじめとするdose dense TC療法に伴う毒性の可能性、通院回数や介入の組み合わせによる費用負担の増大などの可能性もある。

なお、添付文書に記載の副作用は以下の通りである。

1.重大な副作用

＜パクリタキセル＞

　①ショック（0.2%）、アナフィラキシー（0.3%）

②白血球減少等の骨髄抑制

　白血球減少（61.4%）、好中球減少（55.5%）、ヘモグロビン減少（30.7%）、ヘマトクリット値減少（5.0%）、赤血球減少（11.2%）、血小板減少（11.7%）、汎血球減少など

③末梢神経障害（43.8%）、麻痺（0.1％）

④間質性肺炎（0.5%）、肺線維症（頻度不明）

⑤急性呼吸窮迫症候群（0.1％未満）

⑥心筋梗塞、うっ血性心不全（0.1％未満）、心伝導障害（頻度不明）、肺塞栓（0.1％）、血栓性静脈炎（0.4％）脳卒中、肺水腫（0.1％未満）

⑦難聴（0.2％）、耳鳴（0.5％）

⑧消化管壊死（頻度不明）、消化器穿孔、消化管出血（0.1％未満）、消化管潰瘍（0.1％）

⑨重篤な腸炎

　出血性大腸炎（0.1％未満）、偽膜性大腸炎（頻度不明）、虚血性大腸炎（頻度不明）など

⑩腸管閉塞（1.6％）、腸管麻痺（0.1％）

⑪肝機能障害（4.0％）、黄疸（0.1％未満）

⑫膵炎（0.1％未満）

⑬急性腎障害（0.2％）

⑭中毒性表皮壊死融解症、皮膚粘膜眼症候群（頻度不明）

⑮播種性血管内凝固症候群（0.1％）

⑯腫瘍崩壊症候群（頻度不明）

⑰白質脳症（可逆性後白質脳症症候群を含む）（頻度不明）

＜カルボプラチン＞

　①骨髄抑制

　汎血球減少（0.1%未満）、ヘモグロビン減少（40.1%）、赤血球減少（36.1%）、ヘマトクリット値減少（31.7%）、白血球減少（56.4%）、好中球減少（7.4%）、血小板減少（42.7%）、出血（0.1%未満）など

②ショック、アナフィラキシー（0.1%未満）

③間質性肺炎（0.1%）

④急性腎障害（0.1%未満）、ファンコニー症候群（頻度不明）

⑤肝不全、肝機能障害、黄疸（頻度不明）

⑥消化管壊死、消化管穿孔、消化管出血、消化管潰瘍（頻度不明）

⑦出血性腸炎、偽膜性大腸炎（頻度不明）など

⑧麻痺性イレウス（0.1%未満）

⑨脳梗塞（0.1%未満）、肺梗塞（頻度不明）

⑩血栓・塞栓症（頻度不明）

⑪心筋梗塞、うっ血性心不全（頻度不明）

⑫溶血性尿毒症症候群（頻度不明）

⑬急性呼吸窮迫症候群（頻度不明）

⑭播種性血管内凝固症候群（頻度不明）

⑮急性膵炎（頻度不明）

⑯難聴（0.1%未満）

⑰白質脳症（可逆性後白質脳症症候群を含む）（頻度不明）

⑱腫瘍崩壊症候群（頻度不明）

⑲うっ血乳頭、球後視神経炎、皮質盲（頻度不明）

⑳溶血性貧血（頻度不明）

2.その他の副作用

＜パクリタキセル＞

|  | 20％以上 | 5～20％未満 | 5％未満 | 頻度不明 |
| --- | --- | --- | --- | --- |
| 過敏症^注）^ |  | 発疹 | 発赤 |  |
| 循環器 |  | 低血圧 | 不整脈、頻脈、徐脈、期外収縮、高血圧、心悸亢進、心電図異常、心房細動、心室細動、心肥大、狭心症 |  |
| 消化器 | 悪心・嘔吐（35.1%） | 下痢、食欲不振、口内炎、便秘 | 消化不良、鼓腸放屁、胃炎、腹部膨満感、直腸疼痛、嚥下障害、歯肉炎、直腸障害、口唇炎、舌苔、歯肉痛 | 食道炎、粘膜炎、腹水、腸間膜血栓症 |
| 肝臓 |  | AST上昇、Al-P上昇、LDH上昇、ALT上昇 | ビリルビン上昇 |  |
| 泌尿器 |  | 電解質異常、BUN上昇 | クレアチニン上昇、蛋白尿、排尿困難、血尿、尿失禁、尿閉、出血性膀胱炎 |  |
| 皮膚 | 脱毛（45.3%） |  | そう痒、皮膚疾患、爪の障害、皮膚潰瘍、蕁麻疹、皮膚炎、色素沈着、皮膚乾燥、表皮剥離、皮膚腫脹、爪変色 | 斑状丘疹性皮疹、強皮症様変化、亜急性皮膚エリテマトーデス、手足症候群 |
| 精神神経系 |  |  | めまい、不眠、不安、うつ病、傾眠、思考異常、振戦、失神、激越、神経学的疾患、痙攣、運動失調、健忘症、緊張低下、意識障害、寡動、言語障害、緊張亢進、精神症状、譫妄、眼振、不随意運動、嗄声、気分変動 |  |
| 感覚器 |  |  | 味覚倒錯、味覚喪失、視力異常、眼疾患、結膜炎、耳痛、眼痛、霧視、流涙増加、眼精疲労、飛蚊症、眼乾燥、角膜炎、舌異常感、結膜出血、光視症 | 暗点、黄斑浮腫 |
| 呼吸器 |  | 呼吸困難 | 低酸素症、咳増加、喀痰増加、咽頭不快感 |  |
| 全身症状 |  | 無力症、腹痛、倦怠感、頭痛 | 浮腫、疼痛、インフルエンザ様症候群、腹部腫脹、さむけ、体重増加、体重減少 |  |
| 筋骨格 | 関節痛（32.3%）、筋肉痛（28.8%） | 骨痛、背部痛 | 頸部痛、腰痛 | 筋力低下 |
| その他 |  | 発熱、潮紅 | 胸痛、出血、注射部反応、末梢性浮腫、総蛋白減少、アルブミン減少、骨盤痛、発汗、吃逆、口渇、不正出血、無月経、注射部痛、酩酊感、高血糖、低血糖、脱水 | 血栓症 |

注）投与を中止すること

＜カルボプラチン＞

|  | 10％以上 | 1～10％未満 | 1％未満 | 頻度不明 |
| --- | --- | --- | --- | --- |
| 消化器 | 悪心・嘔吐（50.5％）、食欲不振（45.4％） | 下痢、口内炎、腹痛、便秘 | 口渇 |  |
| 腎臓 |  | 血尿、蛋白尿 | 乏尿 |  |
| 過敏症^注）^ |  | 発疹 | 瘙痒感 | 蕁麻疹 |
| 精神神経系 |  | 末梢神経障害（しびれ等）、頭痛 | 耳鳴、聴力低下、視力障害、眩暈、痙攣、異常感覚、味覚異常、神経過敏、不安、不眠 |  |
| 肝臓 | ALT上昇（10.2%） | A S T 上昇、Al-P上昇、ビリルビン上昇、LDH上昇、γ-GTP上昇 |  |  |
| 循環器 |  |  | 心電図異常（期外収縮）、心悸亢進、血圧上昇、血圧低下、不整脈（頻脈、徐脈、心房細動、心房粗動、房室ブロック） |  |
| 電解質 |  | 血清ナトリウム、カリウム、クロール、カルシウム、リン、マグネシウム等の異常 | 抗利尿ホルモン分泌異常症候群 |  |
| 皮膚 | 脱毛（18.3%） |  | 色素沈着、爪の変色、皮膚疾患 |  |
| その他 | 全身倦怠感（18.6%） | 発熱、浮腫 | 疼痛、潮紅、ほてり、胸部不快感、吃逆、注射部位反応（発赤、腫脹、疼痛、壊死、硬結等）、低蛋白血症 | 無力症、尿酸上昇、悪寒、脱水、体重減少、アルブミン低下、呼吸困難 |

注）投与を中止すること

（３）有害事象発生時の研究対象者への対応

　　研究責任医師及び研究分担医師は、有害事象を認めたときは、直ちに適切な処置を行うとともに、診療録ならびに症例報告書に記載する。また、被験薬の投与を中止した場合や、有害事象に対する治療が必要となった場合には、研究対象者にその旨を伝える。有害事象発生時の対応は、原則として保険診療下に行う。

（４）研究計画書等の変更

　　臨床研究を安全に実施する上で必要な情報を常に収集し、新たな安全性情報等が得られた場合、必要に応じて研究計画書及び同意説明文書を変更する。研究計画書や同意説明文書の変更または改訂を行う場合は、認定臨床研究審査委員会の承認を必要とする。実施計画の変更が生じた場合は、以下の届書を厚生労働大臣に提出する。

　① 実施計画事項変更届書（省令様式第二）

　② 変更後の実施計画

　　特定臨床研究の進捗に関する事項の変更については、変更後遅滞なく行う。実施計画の変更を行い、速やかに認定臨床研究審査委員会へ通知するとともに、以下の届書を厚生労働大臣に提出する。

　① 実施計画事項軽微変更届書（省令様式第三）

　② 変更後の実施計画

　　実施計画について以下の軽微な変更を行った場合は、変更後10日以内に、変更内容を認定臨床研究審査委員会に通知する。また、届書（省令様式第三）を厚生労働大臣に提出する。

　① 特定臨床研究に従事する者の氏名、連絡先又は所属する機関の名称の変更であって、当該者又は 当該者の所属する機関の変更を伴わないもの

　② 地域の名称の変更又は地番の変更に伴う変更

　③ 苦情及び問合わせを受け付けるための窓口の変更

　④ 研究責任医師又は研究代表医師の所属する実施医療機関の管理者の氏名の変更

　⑤ 特定臨床研究の実施の可否についての管理者の承認に伴う変更

　⑥ 特定臨床研究の実施状況の確認に関する事項の変更であって、当該特定臨床研究の結果及び監査　の実施の変更を伴わないもの

　⑦ 審査意見業務を行う認定臨床研究審査委員会の名称又は連絡先の変更であって、当該認定臨床研　究審査委員会の変更を伴わないもの

　⑧ ①～⑦に掲げる変更のほか、特定臨床研究の実施の適否及び実施に当たって留意すべき事項に影　響を与えないものとして厚生労働省医政局長が定めるもの

（５）個々の研究対象者における中止基準

【研究中止時の対応】

　　統括管理者、研究責任医師または研究分担医師は、次に挙げる理由で個々の研究対象者について研究継続が不可能と判断した場合には、当該研究対象者についての研究を中止する。その際は、必要に応じて中止の理由を研究対象者に説明する。また、中止後の研究対象者の治療については、研究対象者の不利益とならないよう、誠意を持って対応する。

【中止基準】

　① 研究対象者から研究参加の辞退の申し出や同意の撤回があった場合

　② 本研究全体が中止された場合

　③ 被験薬の有効性が見られなかった場合

　④ 忍容不能な有害事象が発生した場合

　⑤ その他の理由により、研究責任医師または研究分担医師が研究の中止が適当と判断した場合

【被験治療中止時の対応】

　　研究責任医師または研究分担医師は、上記に挙げた理由で個々の研究対象者について被験治療継続ができないと判断した場合には、当該研究対象者についての被験治療を中止する。その際は、必要に応じて中止の理由を研究対象者に説明する。また、中止後の研究対象者の治療については、研究対象者の不利益とならないよう、誠意を持って対応する。

　被験治療中止例のうち、被験薬投与を行った症例については、安全性評価のため観察を継続する。

# １５．記録(データを含む)の取扱い及び保存・破棄の方法（保管期間を含む）

　　統括管理者は、以下の事項に関する関連する記録について、適切に保存する。

　① 本研究の対象者を特定する事項

　② 本研究の対象者に対する診療及び検査に関する事項

　③ 本研究への参加に関する事項

　④ その他、本研究を実施するために必要な事項

　　本研究で収集した情報は、各実施医療機関において当該機関の規程に従い研究終了後5年が経過した日までの間、施錠可能な場所で保存し、その後は個人情報に十分注意して廃棄する。保管する情報からは氏名、住所、生年月日などの直ちに個人を特定できる情報を削除して保管する。

本研究で収集した情報を電子的に保管する場合は、全てのファイルにパスワードを設定し、不正ソフトウェア対策ならびに外部からの不正アクセス防止について適切な対策を講じる。

　　また、対応表は病院情報システム外で保管しない。症例報告書（格納したPC等を含む）と同一の場所に保管しないなど、適切な管理・漏洩防止に最大限努める。

　　統括管理者は、研究等の実施に係わる必須文書（研究計画書、実施計画、本研究の対象者に対する説明及びその同意に係る文書、総括報告書その他の省令の規定により統括管理者及び研究責任医師が作成した文書又はその写し、認定臨床研究審査委員会から受け取った審査意見業務に係る文書、モニタリングに関する文書、研究対象者識別コードリスト、同意書、症例報告書等の控、その他データの信頼性を保証するのに必要な書類または記録文書など）は、各実施医療機関において当該機関の規程に従い研究終了後5年が経過した日までの間、施錠可能な場所で保存し、その後は個人情報に十分注意して廃棄する。試料については、個人識別情報を削除したのちその試料の種類に応じた適切な方法で廃棄し、コンピュータ上にある情報は、コンピュータから専用ソフトを用いて完全に抹消し、紙媒体（資料）はシュレッダーにて裁断し廃棄する。

# １６．原資料等の閲覧に関する事項

　　本研究における原資料とは、以下のものをいう。

① 研究対象者の同意及び情報提供に関する記録

② 診療記録、臨床検査データ及び画像検査フィルム等、症例登録時のデータ、及び症例報告書の元となった記録

統括管理者、研究責任医師及び実施医療機関は、臨床研究に関連するモニタリング、監査、ならびに認定臨床研究審査委員会及び規制当局の調査の際に、原資料などのすべての臨床研究関連記録を直接閲覧に供する。

# １７．定期報告

　　統括管理者は、本研究の実施状況に関する以下の内容について、研究に関する情報がjRCTで公表された日から起算して、1年ごとに、かつ、当該期間満了後2か月以内に実施医療機関の管理者に報告した上で認定臨床研究審査委員会に報告する。統一書式５定期報告書及び別紙様式３定期報告書を用いる。

① 本研究に参加した研究対象者数

② 本研究に係る疾病等の発生状況及びその後の経過

③ 本研究に係る省令又は研究計画書に対する不適合の発生状況及びその後の対応

④ 本研究の安全性及び科学的妥当性についての評価

⑤ 本研究に対する医薬品等製造販売業者等の関与に関する事項

　　また、認定臨床研究審査委員会が意見を述べた日から1か月以内に、以下の内容を別紙様式３を用いて厚生労働大臣に報告する。

① 実施計画に記載されている認定臨床研究審査委員会の名称

② 認定臨床研究審査委員会による本研究の継続の適否

③ 本研究に参加した対象者数

# １８．研究の資金源、研究期間の研究に係る利益相反及び個人の収益等、研究等の研究に係る利益相反に関する状況

　　本研究は、研究責任医師が所属する診療科（岡山大学病院産科婦人科）の研究費で実施する。また、統括管理者は「臨床研究法における利益相反管理ガイダンス」に従って、利益相反を申告し、その審査と承認を得るものとする。また、研究の利益相反と個人の利益相反に変更がないか、定期報告時に確認し、認定臨床研究審査委員会に報告する。

# １９．研究に関する情報公開の方法（研究計画の登録及び研究結果の公表）

　　本研究は、jRCTに登録する。また、本研究で得られた結果は、適切なタイミングでjRCT及び日本婦人科腫瘍学会、日本癌治療学会、American Society of Clinical Oncology等で発表し、Gynecologic Oncology等の専門学術誌で論文として公表する予定である。なお、プロトコールに関しても可能な限り早いタイミングで、プロトコール論文として公表する。

# ２０．研究対象者等からの相談等への対応

　　研究対象者又はその代諾者等及びその関係者からの相談、問合せ、苦情等に対して、統括管理者及び研究責任医師、研究分担医師は適切かつ迅速に対応する。

相談窓口担当者

岡山大学学術研究院医歯薬学域 　周産期医療学講座　長尾 昌二

　住所：〒700-8558　岡山市北区鹿田町2-5-1

　電話番号：086-235-7320（平日：8時～18時）（産科婦人科医局直通）

　　　　　　086-235-7885（平日夜間、休日）（東５階病棟）

苦情相談窓口

岡山大学病院総合患者支援センター　治験・臨床研究相談窓口

連絡先： 086−235−7744（平日8：30～17：00）

E-mail　iscps@okayama-u.ac.jp

# ２１．代諾者等からのインフォームド・コンセントを受ける場合の手順

　本研究では、代諾者は設定しない。

# ２２．インフォームド・アセントを得る手続き（説明事項、説明方法含む）

　本研究では該当しない。

# ２３．緊急かつ明白な生命の危機が生じている状況での研究に関する要件の全てを満たしていることを確認するための手順

　本研究では該当しない。

# ２４．研究対象者等に経済的負担又は謝礼があればその内容

　　本研究に関連する診療・検査等は、すべて保険診療として実施されるため研究対象者には通常の医療と同様に健康保険の自己負担分が発生する。研究に関連して追加の費用負担は生じない。なお、本研究に参加することへの謝礼はない。

# ２５．疾病等及び不具合が発生した場合の対応

（１）疾病等及び不具合の定義

疾病等とは、特定臨床研究の実施に起因するものと疑われる疾病、障害もしくは死亡または感染症ならびにこれらを引き起こしうる不具合をいう。

（２）重篤な疾病等

　特定臨床研究の実施によると疑われる重篤な疾病等は、以下の通り定義する。

１）死亡または死亡につながるおそれのあるもの

２）１）以外の以下の疾病（その他の疾病）

①　治療のために医療機関への入院又は入院期間の延長が必要となるもの

②　障害

③　障害につながるおそれのある疾病等

④　①から③まで並びに死亡及び死亡につながるおそれのある疾病等に準じて重篤であるもの

⑤　後世代における先天性の疾病又は異常

（３）研究との因果関係

全ての疾病と研究実施あるいは被験治療との関係性を、統括管理者・研究責任医師又は研究分担医師が判断する。被験治療開始との時間的関係だけでなく、基礎疾患の経過、合併症、併用薬、研究手順、事故及びその他の外的因子などに起因することも考慮して判断する。

（４）予測性

疾病の予測性は、添付文書及びインタビューフォームに基づいて判断する。有害事象の性質、重症度または頻度が一致しない場合、未知の有害事象とする。予測される有害事象は、14.（2）を参照。

（５）転帰

研究責任医師及び研究分担医師は、発現したすべての疾病に対して、適切な処置、治療を行い、症状が消失するまで又は検査値が基準値若しくは投与前値に復するまで、あるいは医学的に追跡調査の必要がないと研究責任医師及び研究分担医師が判断するまで追跡調査を実施する。有害事象及び不具合の転帰を次のように分類する： 1)回復（症状、所見、臨検値の消失又は回復）、2)軽快（当該事象の軽減）、3)未回復（変化なし～悪化）、4)死亡、5)不明。

（６）研究責任医師及び研究分担医師の責務（重篤な疾病の報告）

① 研究責任医師及び研究分担医師は、特定臨床研究の実施において重篤な疾病等の発生を知った場合には、研究対象者等への説明、治療等、必要な措置を講じなければならない。

② 重篤な疾病を知り得た研究分担医師は、研究責任医師に報告し、報告を受けた研究責任医師は、速やかに統括管理者及び当該実施医療機関の管理者に報告しなければならない。

（７）統括管理者の責務（重篤な疾病の報告）

①統括管理者は、重篤な疾病等の発生を知った時は、厚生労働省令（臨床研究法施行規則）で定めるところ（以下表参照）により、統一書式8を用いて、その旨を当該特定臨床研究の実施計画に記載されている認定臨床研究審査委員会に報告しなければならない。

②統括管理者は、特定臨床研究の実施に起因すると疑われる疾病等の発生に関する事項で、厚生労働省令で定めるもの（以下表参照）を知ったときは、厚生労働省令で定めるところにより、別紙様式第2-1を用いて、その旨を厚生労働大臣に報告しなければならない。

| 予測性 | 疾病等 | 認定臨床研究審査  委員会への報告 | 厚生労働大臣への報告 |
| --- | --- | --- | --- |
| 予測できない | 死亡  （第54条2項1号イ）  死亡につながるおそれ  （第54条2項1号ロ） | 7日以内 | 7日以内 |
|  | 重篤  （第54条2項2号ロ） | 15日以内 | 15日以内 |
|  | 非重篤 | 定期報告時 | ― |
| 予測できる | 死亡  （第54条2項2号イ（1））  死亡につながるおそれ  （第54条2項2号イ(2)） | 15日以内 | ― |
|  | 重篤  （第54条2項3号） | 30日以内 | ― |
|  | 非重篤  （第54条2項5号） | 定期報告時 | ― |

# ２６．健康被害に対する補償の有無及びその内容

　　本研究の実施に伴い、研究対象者に健康被害が発生した場合の補償責任に備え、当研究は臨床研究保険に加入する。万が一、研究計画の不備に起因して発生した健康被害には臨床試験保険から給付を受けることができる。手術・薬剤による健康被害は保険診療内で対応する。

# ２７．不適合報告

　　研究責任医師は、当該臨床研究が法令等又は研究計画書に適合していない状態（「不適合」という。）であると知ったときは、速やかに、統括管理者及び実施医療機関の管理者に報告しなければならない。研究分担医師は、不適合を知ったときは、速やかに研究責任医師に報告しなければならない。統括管理者は、不適合であって、特に重大なものが判明した場合においては、速やかに認定臨床研究審査委員会の意見を聴かなければならない。

　「重大な不適合」とは、臨床研究の対象者の人権や安全性及び研究の進捗や結果の信頼性に影響を及ぼすものをいう。例えば、選択・除外基準や中止基準、併用禁止療法等の不遵守をいい、臨床研究の対象者の緊急の危険を回避するためその他医療上やむを得ない理由により研究計画書に従わなかったものについては含まない。統括管理者は、重大な不適合が発生した場合は、再発防止策を講じ、研究分担医師や当該臨床研究に従事する者に周知するとともに、再発防止の徹底を図ることとする。

委員会への定期報告時に、当該臨床研究に係る法令等又は研究計画書に対する不適合の発生状況及びその後の対応を報告しなければならない。

# ２８．研究の終了、中止

（１）研究の終了

統括管理者は、研究計画書に記載した主たる評価項目に係わるデータの収集を行うための期間が終了した時は、原則として１年以内に主要評価項目報告書を、臨床研究の内容に関する事項として記載した全ての評価項目に係るデータの収集を行うための期間が終了したときには原則として１年以内に総括報告書及びその概要をそれぞれ作成し､認定臨床研究審査委員会に提出する。

なお、主要評価項目報告書及び総括報告書を作成しなければならない時期が同時期になった場合は、総括報告書の作成により主要評価項目報告書の作成をしたものとする。

統括管理者は、主要評価項目報告書又は総括報告書の概要を認定臨床研究審査委員会が意見を述べた日から１ヶ月以内に厚生労働大臣に提出し、jRCTに公表する。厚生労働大臣に総括報告書の概要を提出する際は、研究計画書、説明文書を併せて提出する。

また、統括管理者は、主要評価項目報告書又は総括報告書及びその概要をそれぞれ作成したときは、遅滞なく、これらの内容を研究責任医師に通知するとともに、主要評価項目報告書又は総括報告書の概要について、公表しなければならない。研究責任医師は、当該通知の内容を速やかに実施医療機関の管理者に報告する。

（２）研究の中止

　　統括管理者及び研究責任医師は、以下の事項に該当する場合は研究実施継続の可否を検討する。

１）本研究に使用する薬品、手術機器の品質、安全性、有効性に関する重大な情報が得られたとき。

２）研究対象者のリクルートが困難で予定症例を達成することが到底困難であると判断されたとき。

３）予定症例数または予定期間に達する前に、試験の目的が達成されたとき。

４）認定臨床研究審査委員会により、実施計画等の変更の指示があり、これを受入れることが困難と判断されたとき。

認定臨床研究審査委員会により、中止の勧告あるいは指示があった場合は、研究を中止する。

研究の中止を決定した時は、その旨をその中止の日から10日以内に統一書式11を用いて、当該特定臨床研究の実施計画に記載されている認定臨床研究審査委員会に通知するとともに、様式第四を用いて、厚生労働大臣に届け出る。統括管理者は、中止届を提出した場合であっても、臨床研究が終了するまでの間においては、疾病等報告、定期報告等を行う。また、特定臨床研究が終了するまでの間において、特定臨床研究の進捗状況に関する事項の変更に該当する場合は、実施計画の変更の届出を行う。

# ２９．研究対象者の健康、遺伝的特徴に関する重要な知見が得られる可能性がある場合の研究結果（偶発的所見を含む）の取扱い(研究結果の開示の方針、開示の方法等)

　　研究の実施に伴い、研究対象者の健康に関する重要な知見、またはその他の重要な知見が得られた場合は、研究対象者に知らせることがある。

# ３０．研究に関する業務の一部を委託する場合には、当該業務内容及び委託先の監督方法

　　本研究に関する業務を他の機関に委託することはない。

# ３１．本研究で得られた試料・情報を将来の研究に用いる可能性

　　本研究で得られた情報を将来の研究に用いる可能性があるが、その場合は倫理委員会の承認を得ることとする。

# ３２．モニタリング及び監査の実施体制及び実施手順

（１）モニタリング

　　本研究ではモニタリングを実施する。具体的な手順については、別途モニタリング計画書にて定める。

（２）監査

　　本研究では実施しない。

# ３３．知的財産権、所有権の帰属先

この研究から成果が得られ、知的財産権などが生じる可能性はない。

# ３４．参考資料・文献リスト

1) 子宮頸部　がん種別統計情報　国立研究開発法人国立がん研究センターがん情報サービス ttps://ganjoho.jp/reg_stat/statistics/stat/cancer/17_cervix_uteri.html

2) 厚生労働省　令和3年度「出生に関する統計」の概況https://www.mhlw.go.jp/toukei/saikin/hw/jinkou/tokusyu/syussyo07/index.html

3) 子宮頸がん治療ガイドライン2022年版 日本婦人科腫瘍学会編 金原出版, 東京, 2022

4) Liu CK, Huang KG, Chen MJ, et al. The current trend of fertility preservation inpatients with cervical cancer. Gynecology and Minimally Invasive Therapy 2024;13 :4–9.

5) Manning-Geist B, Grace MA, Sonoda Y. Trachelectomy and fertility-sparing procedures for early-stage cervical cancer: A state of the science review. Gynecol Oncol 2024;181:179–182.

6) Taliento C, Scutiero G, Battello G, et al. Reproductive, obstetrical and oncological outcomes of fertility-sparing treatment for cervical cancer according to the FIGO 2018 staging system: A systematic review. Eur J Surg Oncol 2024;51(1):online ahead of print.

7) Plante M, Kwon JS, Ferguson S, et al. Simple versus radical hysterectomy in women with low-risk cervical cancer. N Engl J Med 2024;390:819–829.

8) Schmeler KM, Pareja R, Lopez BA, et al. **ConCerv: a prospective trial of conservative surgery for low-risk early-stage cervical cancer.** Int J Gynecol Cancer 2021;**31**:1317–1325.

9) Ramirez PT, Robledo KP, Frumovitz M, et al. LACC trial: final analysis on overall survival comparing open versus minimally invasive radical hysterectomy for early-stage cervical cancer. J Clin Oncol 2024;42:2741–2746.

10) Chacon E, Manzour N, Zanagnolo V, et al. **SUCCOR cone study: conization before radical hysterectomy.** Int J Gynecol Cancer 2022;**32**:117–124.

11) Plante M, van Trommel N, Lheureux S, et al. FIGO 2018 stage IB2 (2-4 cm) Cervical cancer treated with Neo-adjuvant chemotherapy followed by fertility Sparing Surgery (CONTESSA); Neo-Adjuvant Chemotherapy and Conservative Surgery in Cervical Cancer to Preserve Fertility (NEOCON-F). A PMHC, DGOG, GCIG/CCRN and multicenter study. Int J Gynecol Cancer 2019 Jun;29(5):969–975.

12) Maneo A, Chiari S, Bonazzi C, Mangioni C. Neoadjuvant chemotherapy and conservative surgery for stage IB1 cervical cancer. Gynecol Oncol 2008;111(3):438–443.

13) Marchiole P, Tigaud JD, Costantini C, et al. Neoadjuvant chemotherapy and vaginal radical trachelectomy for fertility-sparing treatment in women affected by cervical cancer (FIGO stage IB-IIA1). Gynecol Oncol 2011;122(3):484–490.

14) Calvert A.H. et al. Carboplatin dosage : Prospective evaluation of a simple formula based on renal function. J.Clin.Oncol 1989;7:1748–56.

15) Donald W.Conckcroft, Henry Gault. Prediction of Creatinin Clearance form Serum Creatinine:Nephron(1976)16(1):31-41.

16) Kenter GG, Greggi S, Vergote I, et al. Randomized Phase III Study Comparing Neoadjuvant Chemotherapy Followed by Surgery Versus Chemoradiation in Stage IB2-IIB Cervical Cancer: EORTC-55994. J Clin Oncol 2023;41(32):5035–5043.

17) Gupta S, Maheshwari A, Parab P, et al.  Neoadjuvant chemotherapy followed by radical surgery versus concomitant chemotherapy and radiotherapy in patients with stage IB2, IIA, or IIB squamous cervical cancer: A randomized controlled trial. J Clin Oncol. 2018 Jun 1;36(16):1548–1555.

18) Vercellino GF, Piek JMJ, Schneider A, et al. Laparoscopic lymph node dissection should be performed before fertility preserving treatment of patients with cervical cancer. Gynecol Oncol 2012;126(3):325–329.

19) Kim HS, Sardi JE, Katsumata N, et al. Efficacy of neoadjuvant chemotherapy in patients with FIGO stage IB1 to IIA cervical cancer: an international collaborative meta-analysis. Eur J Surg Oncol 2013;39(2):115–124.

20) Schmeler KM, Frumovitz M, Ramirez PT. Conservative management of early stage cervical cancer: is there a role for less radical surgery? Gynecol Oncol 2011;120(3):321–325.

21) Tseng JH, Aloisi A, Sonoda Y, et al. Less versus more radical surgery in stage IB1 cervical cancer: A population-based study of long-term survival. Gynecol Oncol 2018;150(1):44–49.

22) Covens A, Rosen B, Murphy J, et al. How important is removal of the parametrium at surgery for carcinoma of the cervix? Gynecol Oncol 2002;84 (1):145–149.

23) Stegeman M, Louwen M, van der Velden J, et al. The incidence of parametrial tumor involvement in select patients with early cervix cancer is too low to justify parametrectomy. Gynecol Oncol 2007;105 (2):475–480.

24) Tanioka M, Yamaguchi S, Shimada M et al. Cisplatin with dose dense paclitaxel before and after radical hysterectomy for locally advanced cervical cancer: a prospective multicenter phase II trial with a dose-finding study. Med Oncol 2017 Aug;34(8):134.

25) Nagao S, Yamamoto K, Oishi T, et al. Phase II study of a new multidisciplinary therapy using once every 3 weeks carboplatin plus dose -dense weekly paclitaxel before and after radical hysterectomy for locally advanced cervical cancer. Int J Clin Oncol 2020;26(1):207–215.

26) Nagao S, Fujuwara K, Oda T, et al. Combination chemotherapy of docetaxel and carboplatin in advanced or recurrent cervix cancer. A pilot study. Gynecol Oncol 2005;96:805–809.

27) Takekida S, Fujiwara K, Nagao S, et al. Phase II study of combination chemotherapy with docetaxel and carboplatin for locally advanced or recurrent cervical cancer. Int J Gynecol Cancer 2010;20:1563–1568.
