# Supplementary material for: Fertility-sparing surgery with neoadjuvant chemotherapy in early and locally advanced cervical cancer: A clinical protocol
Source: PLoS One. 2026 Jan 13;21(1):e0340963. doi: 10.1371/journal.pone.0340963 (PMC12798975; doi:10.1371/journal.pone.0340963)

**Fertility preservation using neoadjuvant chemotherapy and cervical conization
followed by laparoscopic pelvic lymphadenectomy for FIGO stage IB2-IB3 cervical cancer**

*(CC-NAC C: FepCC)*

**Study Protocol**

Principal Investigator
Shoji Nagao, MD, PhD
Department of Perinatal Medicine,
Okayama University Graduate School of Medicine, Dentistry and Pharmaceutical Sciences
2-5-1 Shikata-cho, Kita-ku, Okayama, 700-8558, Japan
Tel: +81-86-235-7320

Version 1.0: January 1, 2025
Version 1.1: May 29, 2025
Version 2.0: June 17, 2025
Version 3.0: July 2, 2025

# 0. Study Overview

## Objective

The objective of this study is to evaluate the oncological safety and perinatal/gynecological validity of fertility preservation using cervical conization and laparoscopic pelvic lymphadenectomy following neoadjuvant chemotherapy (NAC) in patients with locally advanced cervical cancer (FIGO stage IB2–IB3, 2018 classification: squamous cell carcinoma, adenocarcinoma, or adenosquamous carcinoma) with tumor size greater than 2 cm.

## Primary Endpoint

1) Proportion of patients in whom uterine preservation is achieved. Patients who undergo concurrent chemoradiotherapy (CRT) will not be considered as having achieved uterine preservation.

## Secondary Endpoints

- 2-year recurrence-free survival (RFS) rate
- 2-year overall survival (OS) rate
- Recurrence-free survival duration
- Overall survival duration
- Gynecological outcomes (including quality of life)
- Proportion of patients who resumed menstruation and ovulation
- Proportion of patients who achieved pregnancy
- Proportion of live births, miscarriages, and preterm births
- Type, grade, and incidence of toxicities (classified using CTCAE v5.0)

## Eligible Patients

### Inclusion Criteria

1) Patients clinically diagnosed with stage IB2 or IB3 cervical cancer (FIGO 2018 classification)
2) Histologically confirmed squamous cell carcinoma, adenocarcinoma, or adenosquamous carcinoma
3) Premenopausal patients
4) Patients aged ≤40 years
5) Patients with adequate organ function
6) Patients desiring fertility preservation
7) Patients who have received sufficient explanation, fully understood the study, and voluntarily provided written informed consent

### Exclusion Criteria

- Patients with HPV-independent carcinoma
- Patients with any active concurrent malignancy
- Patients with severe comorbidities
- Patients with hypersensitivity to polyoxyethylene castor oil (Cremophor EL®)-containing agents (e.g., cyclosporine) or hardened castor oil-containing agents (e.g., injectable vitamins)
- Patients with active infections requiring antibiotics
- Pregnant or breastfeeding women, or those who may be pregnant
- Any patient judged inappropriate as a study subject by the principal or sub-investigator

## Intervention

## Study Period

From the date of public disclosure on the jRCT registry following approval by the Certified Review Board, until March 31, 2030.

## Target Sample Size

10 patients

# 1. Title of the Study

Fertility preservation using neoadjuvant chemotherapy and cervical conization followed by laparoscopic pelvic lymphadenectomy for FIGO stage IB2–IB3 cervical cancer (CC-NAC C: FepCC)

# 2. Research Implementation System

This study will be conducted under the following system.

## Principal Investigator and Study Supervisor

Affiliation: Department of Perinatal Medicine, Okayama University Graduate School of Medicine, Dentistry and Pharmaceutical Sciences
Position: Physician
Name: Shoji Nagao
Address: 2-5-1 Shikata-cho, Kita-ku, Okayama 700-8558, Japan
Phone: +81-86-235-7320 (direct line to department)

## Monitoring Supervisor

Affiliation: Department of General Medicine, Southwestern Okayama (Kasaoka),
Position: Physician
Name: Takashi Mitsui
Phone: +81-86-235-7320 (Weekdays 8:00–18:00, direct line to department)

# 3. Background, Objective, and Significance

## Background

### (1) Epidemiology of Cervical Cancer

In Japan, approximately 10,000 women are newly diagnosed with cervical cancer each year, and around 3,000 die from the disease. Persistent infection with human papillomavirus (HPV) in the cervix is the primary cause, and the disease predominantly affects young women. Among women in their 30s, cervical cancer is the second most common malignancy after breast cancer, and about 42% of cases occur in women under the age of 45, i.e., those of reproductive age.

Meanwhile, the average maternal age at first delivery in Japan has risen from 25.7 years in 1975 to 30.9 years in 2021. Hysterectomy remains the mainstay treatment for early-stage and locally advanced cervical cancer; however, this procedure deprives women of childbearing age of fertility and has a profound impact on pregnancy and childbirth. Although the spread of HPV vaccination is expected to reduce cervical cancer incidence in the future, its effect in Japan has not yet been confirmed. Consequently, the demand for fertility preservation in young women with early or locally advanced cervical cancer remains high.

### (2) Current Status of Fertility-Sparing Surgery for Cervical Cancer

According to the 2022 edition of the Cervical Cancer Treatment Guidelines, fertility-sparing surgery is recommended for women of reproductive age with cervical cancer who wish to preserve fertility. For stage IA1, cervical conization is recommended; for stage IA2 and IB1, radical trachelectomy is recommended as a method of uterine preservation. However, for stage IB2 and IB3, where the tumor diameter exceeds 2 cm, radical hysterectomy is recommended even if the tumor is confined to the cervix, and fertility-sparing surgery is not indicated.

On the other hand, although radical trachelectomy has been shown to be oncologically safe for cervical cancer confined to the cervix with tumors ≤2 cm, it is associated with a high incidence of complications such as miscarriage, preterm delivery, and bleeding, thereby limiting perinatal outcomes.

### (3) International Approaches

The SHAPE and ConCerv trials demonstrated that for early-stage cervical cancer with tumors ≤2 cm, simple hysterectomy or cervical conization with pelvic lymphadenectomy achieves outcomes equivalent to radical hysterectomy. Based on these results, there is now a global consensus that less radical surgery, such as laparoscopic simple hysterectomy or cervical conization, is an acceptable option for IB1 or smaller tumors.

Furthermore, the LACC and SUCCOR cone trials also demonstrated that in cases with tumors ≤2 cm where negative margins were achieved after conization, laparoscopic simple hysterectomy provides excellent prognosis. Thus, tumor size ≤2 cm (IB1 or smaller) is considered a key criterion for applying less radical surgery for cervical cancer.

Currently, in Western countries, there is increasing interest in using neoadjuvant chemotherapy (NAC) to enable fertility preservation in young patients with cervical cancer whose tumors exceed 2 cm (stage IB2 or higher). For instance, Maneo et al. treated 51 patients (≤3 cm, ≤40 years, stage IB1) with NAC (cisplatin 75 mg/m2 + paclitaxel 175 mg/m2 + ifosfamide 5 g/m2, 3 cycles) followed by laparoscopic pelvic lymphadenectomy and conization. Radical hysterectomy was performed only if residual disease was found on frozen section. Among these, 21/51 patients (41%) had no residual tumor and underwent conization alone. None of these 21 relapsed, and 6 of 9 who attempted pregnancy conceived.

Similarly, Marchiole et al. treated seven patients with stage IB–IIA1 cervical cancer (tumors 30–45 mm) with NAC (cisplatin 75 mg/m2 + paclitaxel 175 mg/m2 + ifosfamide 5 g/m2, 3 cycles) followed by laparoscopic pelvic lymphadenectomy and radical vaginal trachelectomy. One patient had parametrial involvement requiring additional brachytherapy, but no recurrences were observed after 22 months of follow-up. These results suggest that, in carefully selected cases where NAC achieves tumor shrinkage to ≤2 cm, fertility-sparing conservative surgery including conization may be feasible.

However, despite numerous case reports and small case series describing conservative surgery after NAC in cervical cancer downsized to ≤2 cm, no large-scale, consolidated studies have yet been conducted.

### (4) Significance of Investigating Conization after NAC in Fertility-Sparing Patients

If oncological safety can be confirmed for cervical cancer patients with tumors >2 cm undergoing cervical conization after NAC, this approach could significantly expand the indication for fertility preservation while simultaneously avoiding perinatal complications. This strategy is expected to bring substantial benefits to patients with locally advanced cervical cancer who wish to preserve fertility.

## Objective of the Study

The purpose of this study is to evaluate the oncological safety and perinatal/gynecological validity of fertility preservation using cervical conization and laparoscopic pelvic lymphadenectomy following NAC in patients with locally advanced cervical cancer (FIGO stage IB2–IB3, 2018 classification: squamous cell carcinoma, adenocarcinoma, or adenosquamous carcinoma) with tumors >2 cm. If the safety and usefulness of this strategy are confirmed, it may provide a new treatment option for young women with stage IB2–IB3 cervical cancer who desire fertility preservation.

# 4. Methods

## (1) Study Type and Design

This study is a single-institution, prospective interventional study. It is designed as an open-label, single-arm, phase II trial.

## (2) Study Outline (see flow chart)

Written informed consent will be obtained, and patients who meet all inclusion criteria and none of the exclusion criteria will be considered eligible. After screening tests and confirmation of eligibility, patients will be registered and receive dose-dense TC chemotherapy.

Among patients who successfully complete three cycles of dose-dense TC chemotherapy, those with pelvic MRI (as well as pelvic exam, colposcopy, and transvaginal ultrasound) showing a maximum tumor diameter ≤2 cm and no new lesions, and with PET-CT or pelvic-to-neck CT confirming no new lesions, will undergo cervical conization.

Patients who drop out will undergo open radical hysterectomy. If postoperative pathological evaluation of conization specimens confirms tumor diameter ≤2 cm and negative surgical margins, laparoscopic pelvic lymphadenectomy will be performed. If lymph node metastasis is detected, concurrent chemoradiotherapy with cisplatin (biweekly) will be administered.

All registered patients, including those who drop out, will be followed up for two years.

The primary endpoint is the proportion of patients who achieve uterine preservation. Secondary endpoints include: 2-year recurrence-free survival (RFS), 2-year overall survival (OS), RFS duration, OS duration, gynecological outcomes (including quality of life), proportions of patients resuming menstruation and ovulation, proportion achieving pregnancy, live birth rate, and type, grade, and incidence of toxicities (CTCAE v5.0).

If, after the enrollment of 5 patients, 2 or fewer achieve uterine preservation, the feasibility of continuing the study will be re-evaluated.

## (3) Expected Participation Period per Patient

Total: 130 weeks (approximately 2.5 years)
- Screening period: 4 weeks
- Intervention period: 22 weeks (NAC: 12 weeks, conization: 5 weeks, pelvic lymphadenectomy: 5 weeks)
- Follow-up period: 104 weeks (2 years)

## (4) Investigational Drugs and Devices

### Paclitaxel

Generic name: Paclitaxel
Formulation: Paclitaxel injection 100 mg/16.7 mL (manufactured by Nippon Kayaku Co., Ltd.)
Description: Colorless to pale yellow clear viscous liquid, pH 3.0–7.0 (10% aqueous solution)
Pharmacological classification: Antineoplastic agent
Indication: Advanced or recurrent cervical cancer
Dosage: 135 mg/m² IV infusion over 24 hours, repeated every 3 weeks
Storage: Room temperature, in box after opening

### Carboplatin

Generic name: Carboplatin
Formulation: Carboplatin IV infusion 150 mg/15 mL (manufactured by Viatris Healthcare GK and Nippon Kayaku Co., Ltd.)
Description: Colorless to pale yellow clear liquid, pH 5.5–6.5
Pharmacological classification: Antineoplastic agent
Indication: Cervical cancer
Dosage: 300–400 mg/m² IV infusion every 4 weeks, adjusted based on age, disease, and condition
Storage: Protect from light and heat, store in original box; may precipitate if refrigerated

## (5) Intervention Details

### Intervention 1: Neoadjuvant Chemotherapy (NAC)

The NAC regimen will consist of dose-dense TC therapy: paclitaxel 80 mg/m² (days 1, 8, and 15, IV infusion) plus carboplatin AUC = 6 (day 1, IV infusion), repeated every 21 days as one cycle.

#### 1. Calculation of Body Surface Area (BSA)

The DuBois formula will be used:
BSA (m²) = Body Weight^0.425 × Height^0.725 × 71.84 / 10,000
where Body Weight (kg), Height (cm).
The maximum BSA value for dosing will be capped at 2.0 m².

#### 2. Calculation of Paclitaxel Dose

The dose of paclitaxel will be calculated based on the BSA derived from the DuBois formula.

#### 3. Calculation of Carboplatin Dose

The Calvert formula will be used:
Carboplatin dose (mg) = Target AUC × (GFR + 25)

1) In this trial, GFR will be assumed equivalent to creatinine clearance (Ccr).
2) Ccr will be calculated using the Cockcroft–Gault equation:
 Ccr = 0.85 × (140 – age) × BW / (72 × serum creatinine) × 1.73
 where BW (kg), serum creatinine (mg/dL).
3) If serum creatinine is <0.6 mg/dL, it will be corrected to 0.6 mg/dL.
4) If no new urinary obstruction or CTCAE grade ≥2 renal impairment occurs, recalculation in subsequent cycles is not required.
5) The maximum carboplatin dose will be capped at 1000 mg per body.

#### 4. Administration of Paclitaxel

1) Following premedication, the calculated dose will be diluted in 250 mL of 5% glucose or normal saline and administered via a dedicated line over 1 hour.
2) Administered on days 1, 8, and 15 of each cycle.

#### 5. Administration of Carboplatin

1) Administered immediately after paclitaxel infusion. The calculated dose will be diluted in 250 mL of 5% glucose or normal saline and infused over 1 hour.
2) Administered on day 1 of each cycle.

#### 6. Criteria for Initiating Treatment

Cycle 1 Day 1: All inclusion laboratory and clinical criteria must be confirmed within 2 weeks prior to initiation.

Cycle 1 Day 8, 15: Within 2 days prior to dosing, the following must be met:
- Neutrophils ≥ 500/mm³
- Platelets ≥ 50,000/mm³
If criteria are not met, dosing may be delayed up to 3 weeks; beyond 3 weeks, treatment will be discontinued.

Cycle 2+ Day 1: Within 2 days prior to dosing:
- Neutrophils ≥ 1,000/mm³
- Platelets ≥ 75,000/mm³
- Non-hematologic toxicities ≤ Grade 1 (except alopecia, fatigue, nausea, constipation)

Cycle 2+ Day 8, 15: Within 2 days prior to dosing:
- Neutrophils ≥ 500/mm³
- Platelets ≥ 50,000/mm³

#### 7. Dose Reduction Criteria

If any of the reduction criteria are met, the dose for the next cycle will be reduced by 1 level. If criteria are met again, reduce an additional level. Dose reduction is limited to a maximum of 2 steps. If criteria persist after two reductions, NAC will be discontinued. Re-escalation is not allowed.

Reduction levels:
Level 0: Paclitaxel 80 mg/m², Carboplatin AUC 6
Level -1: Paclitaxel 70 mg/m², Carboplatin AUC 5
Level -2: Paclitaxel 60 mg/m², Carboplatin AUC 4
Level -3: Discontinue both agents

### 8. Management of Adverse Events and Treatment Modifications

#### Criteria for Termination or Discontinuation of Dose-Dense TC Therapy

1) Completion Criteria:
- In principle, dose-dense TC therapy will be completed after 3 cycles.

2) Discontinuation Criteria:
- Delay exceeding 3 weeks
- Occurrence of Grade 4 non-hematologic toxicity
- Requirement for Level -3 dose reduction
- Patient request to discontinue therapy
- Death during treatment
- Disease progression or recurrence after initiation
- Investigator’s judgment that continuation is not appropriate for other reasons

### 9. Concomitant and Supportive Therapies

1) G-CSF: Prophylactic use is not permitted. Therapeutic use may be considered according to ASCO guidelines in the following situations:
- Neutrophil count <1,000/mm³ with fever ≥38°C
- Neutrophil count <500/mm³
- History of such events in prior cycles with neutropenia <1,000/mm³
Dosage: 50 µg/m² subcutaneously once daily until recovery.

2) Anemia: Iron supplementation or transfusion as necessary.

3) Antiemetics: Preventive use of 5-HT3 antagonists and other antiemetics is recommended.

4) Anti-allergy medications: Steroids and antihistamines for prevention of paclitaxel-related hypersensitivity.

5) Premedication: Administer diphenhydramine 50 mg orally, dexamethasone 20 mg IV, and ranitidine 50 mg IV 30 minutes before paclitaxel infusion (short premedication regimen). If no hypersensitivity occurs in cycle 1, subsequent dexamethasone doses may be reduced.

6) Other allowed therapies: Concomitant medications for complications or adverse events (e.g., antibiotics, analgesics, transfusions) and symptomatic treatment for peripheral neuropathy are permitted.

7) Prohibited therapies: Any other anticancer drugs, immunotherapy, hormonal therapy, radiotherapy, or investigational drugs are prohibited until the completion/discontinuation of dose-dense TC therapy.

### 10. Evaluation Methods

Tumor size will be evaluated after each cycle using pelvic examination, colposcopy, transvaginal ultrasound, and pelvic MRI.

### Intervention 2: Cervical Conization

Eligibility criteria for conization:
1) Maximum tumor diameter ≤2 cm confirmed by colposcopy, transvaginal ultrasound, and pelvic MRI.
2) ≥3 mm of normal cervical stroma remaining and <50% stromal invasion, with no new lesions.

Surgical procedure:
- General or spinal anesthesia may be used.
- After ligating the descending branches of the uterine artery at 3 and 9 o’clock positions, mark the cervix circumferentially ≥5 mm from the tumor with silk sutures and resect while applying traction.
- Resect the cervix to achieve complete tumor removal but do not open the vesicouterine peritoneum or pouch of Douglas.
- If the tumor is not visible (complete response to NAC), excise the cervix with a depth of approximately 2 cm.

If postoperative pathology shows residual tumor ≤2 cm with positive margins or unevaluable margins (due to cauterization), repeat conization will be considered. If conization is performed twice, tumor size and stromal invasion depth will be summed for risk assessment.

If pathology shows tumor diameter >2 cm or stromal invasion >10 mm, open radical hysterectomy will be performed. If new lesions are found, radiotherapy (including CRT) or chemotherapy will be considered.

### Intervention 3: Laparoscopic Pelvic Lymphadenectomy

Eligibility criteria based on conization pathology:
1) Tumor diameter ≤2 cm
2) Stromal invasion ≤10 mm
3) Negative surgical margins
4) No lymphovascular invasion

Patients meeting all these conditions will undergo laparoscopic pelvic lymphadenectomy.

Surgical considerations:
- Surgery will be performed in collaboration between a board-certified laparoscopic surgeon of the Japan Society of Gynecologic and Obstetric Endoscopy and Minimally Invasive Therapy and a board-certified gynecologic oncologist of the Japan Society of Gynecologic Oncology, or by a gynecologic oncologist with sufficient laparoscopic expertise.
- Device choice, port placement, and dissection technique will not be restricted.
- Lymphadenectomy will cover all regional lymph nodes, including common iliac, external iliac, internal iliac, obturator, sacral, cardinal ligament, and suprainguinal nodes, following the Japanese Classification of Cervical Carcinoma.
- To ensure standardization and adequacy, a target of 20 lymph nodes (pathologically confirmed) will be set.
- Post-dissection, photographs of the bladder and rectal pararectal spaces demonstrating identification of levator ani, obturator internus, coccygeus, hypogastric nerve, and internal iliac vein will be archived to assure surgical quality.

Exclusion criteria for Intervention 3:
1) Intraoperative detection of peritoneal dissemination or ovarian metastasis (surgery discontinued).
2) Enlarged nodes submitted for frozen pathology: if positive, radical hysterectomy will be performed via laparotomy; if multiple positive nodes are found, surgery will be discontinued, and further treatment will follow standard practice.
3) If postoperative pathology reveals lymph node metastasis, radiotherapy (including CRT) will be administered.

## (6) Management of Investigational Drugs

All drugs used in this study are already approved. Proper storage and handling will be maintained in accordance with approved indications and package inserts.

## (7) Rules on Concomitant Therapies

1) Allowed: Supportive therapies consistent with routine clinical care, including preventive management for chemotherapy-related toxicities.
2) Prohibited: Any anticancer therapy including radiotherapy, chemotherapy, immunotherapy, and hormonal therapy.

## (8) Patient Registration

The principal investigator or sub-investigator will register patients according to the following procedures:
1) Confirm eligibility (all inclusion criteria met, no exclusion criteria) and obtain written informed consent.
2) Prepare a subject identification code list linking consent date and ID, stored by the principal investigator.
3) Any withdrawal of consent, discontinuation, or dropout must be promptly reported to the principal investigator.

## (9) Post-Study Care

After study completion, the principal investigator will provide patients with the most appropriate medical care, including consideration of the study results.

# 5. Endpoints

## Primary Endpoint

1) Proportion of patients in whom uterine preservation is achieved.
Patients who undergo concurrent chemoradiotherapy (CRT) will not be considered as having achieved uterine preservation.

## Secondary Endpoints

1) 2-year recurrence-free survival (RFS) rate
2) 2-year overall survival (OS) rate
3) Recurrence-free survival duration
4) Overall survival duration
5) Gynecological outcomes, including quality of life (FACT-Cx, FSFI, HADS questionnaires)
6) Proportion of patients who resumed menstruation and ovulation
7) Pregnancy rate
8) Live birth rate, miscarriage rate, preterm birth rate
9) Type, grade, and incidence of toxicities (evaluated by CTCAE v5.0)

# 6. Follow-up and Observation Items

Patients will be followed for two years after completion of intervention to evaluate oncological safety and fertility outcomes.

## Oncological Assessment

- Pelvic examination and cytology: every 3 months
- Pelvic MRI: every 6 months
- PET-CT or chest–abdomen–pelvis CT: annually or when recurrence is suspected
- Recurrence will be assessed according to RECIST version 1.1 criteria.

## Gynecological and Perinatal Assessment

- Gynecologic questionnaires (FACT-Cx, FSFI, HADS): every 6 months
- Menstruation and ovulation will be monitored using basal body temperature charts and patient reports.
- Pregnancy, delivery outcomes, miscarriage, and preterm birth will be recorded through patient interviews.

# 7. Safety Evaluation

All adverse events will be documented and graded using CTCAE version 5.0. Serious adverse events (SAEs) will be reported promptly to the Certified Review Board (CRB) and the hospital ethics committee. Safety will be monitored throughout the study period by the monitoring supervisor. If necessary, the study protocol may be revised or the study terminated early based on safety assessments.

# 8. Study Schedule

The planned study period is from the date of public disclosure on the jRCT registry following approval by the Certified Review Board until March 31, 2030.

Patient enrollment is expected to take approximately 5 years, and each patient will be followed for 2 years after completion of the intervention.

# 9. Target Sample Size

A total of 10 patients will be enrolled. This number is considered feasible given the rarity of eligible patients and the exploratory nature of the study.

# 10. Statistical Analysis

## Analysis Set

- Full Analysis Set (FAS): All patients who received at least one dose of the investigational treatment and for whom efficacy and safety data are available.
- Per Protocol Set (PPS): Patients in the FAS who did not have major protocol deviations.
- Safety Analysis Set (SAF): All patients who received at least one dose of the investigational treatment.

## Primary Endpoint Analysis

The uterine preservation rate will be calculated with its 95% confidence interval. An exact binomial test will be applied to evaluate whether the uterine preservation rate exceeds a threshold of 15%.

## Secondary Endpoint Analysis

- Recurrence-free survival (RFS) and overall survival (OS) will be estimated using the Kaplan–Meier method.
- The 2-year RFS rate and 2-year OS rate will be calculated with their 95% confidence intervals.
- Gynecologic and perinatal outcomes will be summarized descriptively.
- Adverse events will be tabulated by type, grade, and incidence (CTCAE v5.0).

# 11. Ethical Considerations

This study will be conducted in accordance with the Declaration of Helsinki, the Clinical Trials Act of Japan, and relevant ethical guidelines for medical research involving human subjects. The protocol and informed consent documents will be reviewed and approved by the Certified Review Board (CRB) of Okayama University Hospital before study initiation.

Written informed consent will be obtained from all participants prior to enrollment. Patients will be informed that participation is voluntary and that they may withdraw consent at any time without disadvantage to future treatment.

# 12. Monitoring and Auditing

Monitoring will be conducted by the monitoring supervisor to ensure that the study is carried out in compliance with the protocol, ethical guidelines, and relevant regulations. The monitor will periodically review case report forms (CRFs), source documents, and consent forms, and will confirm the accuracy and reliability of the collected data.

If deemed necessary, an audit may be conducted by an independent auditing body authorized by Okayama University Hospital. The audit will evaluate whether the study is conducted in accordance with the protocol, laws, and guidelines, and whether data are properly recorded and reported.

# 13. Data Management

All study data will be collected using paper-based case report forms (CRFs). The investigator will ensure that the data are accurate, complete, and recorded in a timely manner. Personal identifiers will be removed, and each subject will be assigned a unique study identification code. The link between the identification code and personal information will be securely stored by the principal investigator.

After completion of the study, the CRFs and all study-related documents will be retained for 5 years at the study site. Access to these documents will be restricted to authorized personnel only.

# 14. Funding and Conflict of Interest

This study is supported by research funding from Okayama University. The investigators declare no conflicts of interest related to this study.

# 15. Publication Plan

The results of this study will be presented at scientific conferences and published in peer-reviewed journals. Authorship will follow the ICMJE (International Committee of Medical Journal Editors) guidelines.

# 16. References

1. Tewari KS. Cervical cancer. N Engl J Med. 2025;392(1):56–71. doi:10.1056/NEJMra2404457.

2. National Cancer Center Japan. Cancer statistics. https://ganjoho.jp (Accessed July 2025).

3. Plante M, Kwon JS, Ferguson S, et al. Simple versus radical hysterectomy in women with low-risk cervical cancer. N Engl J Med. 2024;390(9):819–29. doi:10.1056/NEJMoa2308900.

4. Schmeler KM, Pareja R, Lopez Blanco A, et al. ConCerv: a prospective trial of conservative surgery for low-risk early-stage cervical cancer. Int J Gynecol Cancer. 2021;31(9):1317–25. doi:10.1136/ijgc-2021-002921.

5. Gupta S, Maheshwari A, Parab P, et al. Neoadjuvant chemotherapy followed by radical surgery versus concomitant chemotherapy and radiotherapy in patients with stage IB2, IIA, or IIB squamous cervical cancer: a randomized controlled trial. J Clin Oncol. 2018;36(16):1548–55. doi:10.1200/JCO.2017.75.9985.

6. Maneo A, Chiari S, Bonazzi C, et al. Neoadjuvant chemotherapy and conservative surgery for stage IB1 cervical cancer. Gynecol Oncol. 2008;111(3):438–43. doi:10.1016/j.ygyno.2008.08.013.

7. Marchiole P, Tigaud JD, Costantini S, et al. Neoadjuvant chemotherapy and vaginal radical trachelectomy for fertility sparing treatment in women with stage IB–IIA cervical cancer: oncologic and obstetric outcomes. Gynecol Oncol. 2011;122(3):484–90. doi:10.1016/j.ygyno.2011.05.027.

8. Ramirez PT, Frumovitz M, Pareja R, et al. Minimally invasive versus abdominal radical hysterectomy for cervical cancer. N Engl J Med. 2018;379(20):1895–904. doi:10.1056/NEJMoa1806395.

9. Chiva L, Zanagnolo V, Querleu D, et al. SUCCOR study: an international European cohort observational study comparing minimally invasive surgery versus open abdominal radical hysterectomy in patients with stage IB1 cervical cancer. J Clin Oncol. 2020;38(31):3567–3577. doi:10.1200/JCO.20.00581.

10. Matsuo K, Machida H, Mandelbaum RS, et al. Validation of the revised 2018 FIGO staging system of cervical cancer. Gynecol Oncol. 2019;152(1):87–93. doi:10.1016/j.ygyno.2018.10.026.

Figure 1. Study intervention flow chart


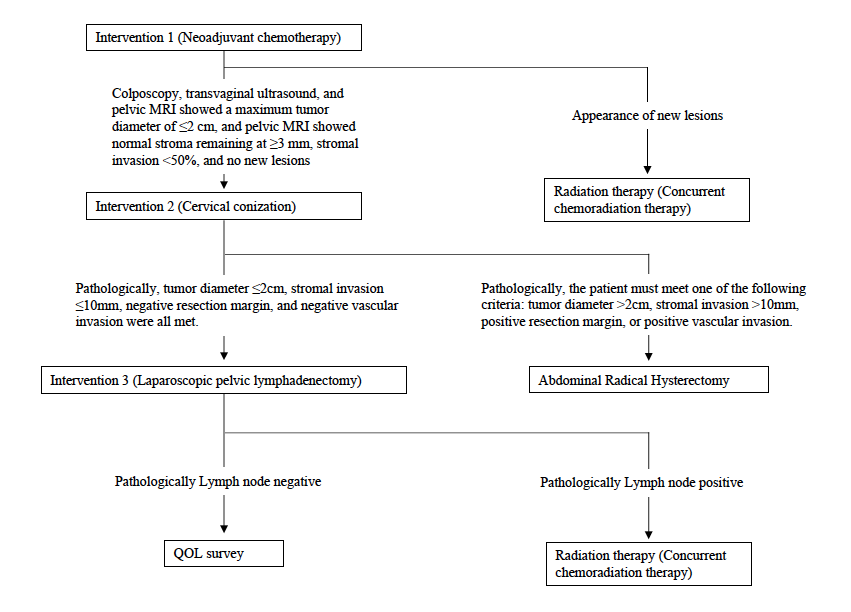


Figure 2. Study design schema


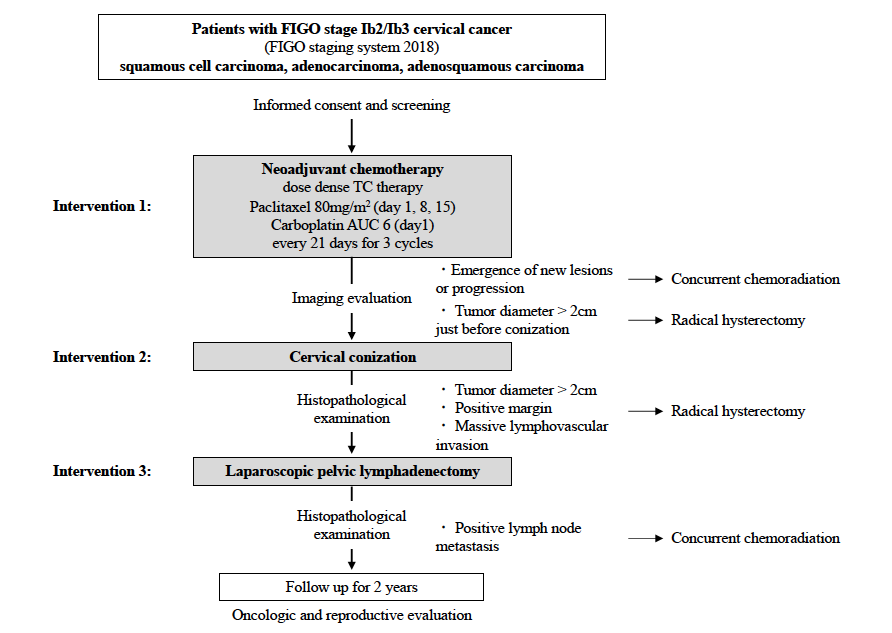

Supplement: S3 File — (DOCX) [file pone.0340963.s003.docx]
