# Supplementary material for: Fertility-sparing surgery with neoadjuvant chemotherapy in early and locally advanced cervical cancer: A clinical protocol
Source: PLoS One. 2026 Jan 13;21(1):e0340963. doi: 10.1371/journal.pone.0340963 (PMC12798975; doi:10.1371/journal.pone.0340963)
Supplement: S4 File — (DOCX) [file pone.0340963.s004.docx]

**患者の皆様へ**

**「妊孕性温存を希望する子宮頸癌IB2-IB3期に対する**

**主治療前化学療法を用いた縮小手術」**

**についてのご説明**

**研究責任医師**

**岡山大学学術研究院医歯薬学域　周産期医療学講座**

**職名：****医師　　氏名：長尾　昌ニ**

**はじめに**

病気の原因の解明や、予防・診断・治療方法の改善などのために、人を対象として行われる研究を「臨床研究」と言います。臨床研究は評価が十分定まっていない新たな診断法や治療方針の有効性や安全性の確立のために行われるもので、通常の診療とは異なり、研究的な性格を伴います。臨床研究から得られた情報は、あなたと同じ病気の患者さんの治療における貴重な情報の一つとなり、これまでの臨床研究の成果が積み重なって現代の医療が成り立っています。このように、医療をさらに進歩・発展させ、より効果的で安全な医療を行うために、臨床研究は欠かせません。そして、臨床研究を行うには、多くの患者さんのご理解とご協力が必要です。この度、私たちはあなたにこれから説明する臨床研究に参加していただきたいと考えています。

なお、この研究は実際の診療に携わる医師が医学的必要性・重要性を考慮して、立案・計画して行うものであり、製薬会社が行う新薬の安全性・有効性を調べ、厚生労働省の承認を得るためのいわゆる「治験」ではありません。また、この研究で行われる医療行為や検査は現時点での標準的な診療ではないものが含まれ、その効果はこれから評価されるものであることをご理解ください。

この研究に参加されるかどうかは、あなたの自由意思で決めて下さい。参加されなくてもあなたが不利益を被ることはありません。説明の中には少し難しい部分もありますが、よくお読みになり、わからない点や不安な点がございましたら、遠慮なく研究責任医師または研究分担医師にお尋ねください。

ご参加いただける場合は、別紙の「同意書」にご署名の上、担当医師にお渡しください。

**１．当該研究の実施について研究機関の長の許可を受けていることについて**

臨床研究を行うためには、研究を行う目的と研究を行う方法が正しく考えられているか、また、参加いただく患者さんの人権が保護され、安全性が確保される内容になっているか、などについて、倫理的・科学的な側面からの審査を受けることが義務付けられています。

この臨床研究については、臨床研究審査委員会にて承認を得ており、当院の管理者（病院長）が実施の許可をしております。また、厚生労働大臣に実施計画を提出し、「臨床研究法」と関連する法令等を遵守して実施します。

＊臨床研究審査委員会

この研究を実施することの適否などについては、以下の委員会により臨床研究が倫理的、科学的に妥当であるかどうかを審査されています。

| 審査委員会の種類 | 岡山大学臨床研究審査委員会 |
| --- | --- |
| 設置者の名称 | 岡山大学学長　那須　保友 |
| 所在地 | 岡山県岡山市北区鹿田町二丁目５番１号 |
| 認定番号 | CRB6180001 |
| 認定日 | 2018年3月30日 |

この臨床研究審査委員会の手順書、委員の名簿、委員会の審議概要などは、ホームページ（URL: https://www.hsc.okayama-u.ac.jp/ethics/nintei/）において一般に公開しており、自由に閲覧することができます。

**２．研究の実施体制について**

この研究は以下の体制で行います。

【統括管理者・研究責任医師】

　　所属：岡山大学学術研究院医歯薬学域　周産期医療学講座

職名：教授　氏名：長尾 昌二

　　住所：〒700-8558　岡山県岡山市北区鹿田町2-5-1

　　電話番号：086-235-7320（平日：8時～18時）（医局直通）

　　　　　　　086-235-7885（平日夜間、休日）（東５階病棟）

**３．この研究の背景、目的、意義**

**１）　あなたの病気について**

　あなたは、子宮頸部に悪性腫瘍を認め、子宮頸癌IB2期（腫瘍のサイズが2cmを超えて4cmまでの場合）あるいはIB3期（腫瘍のサイズが4cmを超えている場合）と診断されています。ここまでの診察、検査では、子宮頸部以外の部位には明らかな病変は確認されていません。適切な治療により、80%から90%程度の治癒が見込まれます。

**２）　あなたの病気に対する標準的な治療法について**

　腫瘍径が２cmを超えていて、かつリンパ節転移を認めない子宮頸癌ⅠB2、ⅠB3期の標準治療は広汎性子宮全摘術（子宮とその周囲の組織を一括して広く摘出する手術で、再発のリスクに応じて術後に放射線治療を追加する場合があります）、あるいは放射線治療（IB3期では同時化学放射線療法）です。手術を選択した場合には、周囲のリンパ組織なども含めて子宮を摘出しますので、その後の妊孕性（妊娠の可能性）は失われます。また、放射線治療を選択した場合にも、放射線の照射により卵巣や子宮内膜の機能が失われますので、やはりその後の妊孕性は失われます。

**３）　この研究の目的、背景、意義**

　子宮頸がん治療ガイドライン2022年版によると、治療終了後の妊娠を希望する初期子宮頸癌の患者さんに対し、広汎性子宮頸部摘出術（子宮頸部を周囲の組織やリンパ節も含めて広範囲に切除する手術）の選択が可能とされています。しかし、広汎性子宮頸部摘出術後には子宮頸部がわずかしか残らず、その脆弱性のため、流早産が多く、妊娠に至っても満期まで継続することは難しいことがわかってきています。しかも、その対象は、転移がなく、かつ腫瘍のサイズが2cm以下の子宮頸癌（IA2期あるいはIB1期）の患者さんに限定されており、腫瘍のサイズが2cmを超えるIB2期やIB3期の患者さんには適用されません。

　近年、一定の条件の腫瘍径2cm以下の子宮頸癌（IA2期あるいはIB1期）の患者さんにおいては、広汎性子宮全摘術や広汎性子宮頸部摘出術のような広範囲に子宮頸部を摘出する手術ではなく、子宮頸部円錐切除術（経腟的に子宮頸部を切除する手術であり比較的しっかりと子宮頸部が温存される）および腹腔鏡下のリンパ節郭清術（腹腔鏡を用いて骨盤リンパ節を摘出する手術で、開腹手術と比ベて侵襲が少ない）でも同等の治療効果が期待できることがわかってきました。この場合には、子宮頸部の強度が維持できるため、広汎性子宮頸部摘出術のような妊娠中のトラブルの起こる可能性が低くなることが期待でき、広く行われるようになってきています。

　一方、手術前の抗がん剤投与（主治療前化学療法：NACといいます）によって、9割を超える患者さんにおいて腫瘍が縮小することが報告されています（広汎性子宮全摘術や放射線治療と比較して予後を改善する効果は確認されていません）。当院ではこれまで多くのIB3期以上の子宮頸癌の患者さんにNAC後の広汎性子宮全摘術を行っており、腫瘍の縮小によって手術の安全性の向上、術後の放射線治療の省略が可能になっています。NACではdose dense TC療法という抗がん剤治療を行います。これは、異なる作用の2種類の抗がん剤、パクリタキセル(Paclitaxel)とカルボプラチン(Carboplatin)を組み合わせた治療法です。3週間を1つのサイクルとして、1日目に2種類の抗がん剤（パクリタキセルとカルボプラチン）を投与し、8日目と15日目にはパクリタキセルのみを投与します。手術までにこの治療を3サイクル行います。そこで、NACを行って腫瘍を縮小させれば、治療終了後の妊娠を希望する子宮頸癌の患者さんに円錐切除術＋腹腔鏡下骨盤リンパ節郭清術による子宮の温存が可能になると考えました。そうすれば、これまで子宮温存の対象になっていなかったIB2期、IB3期の患者さんの子宮を温存し、かつ妊娠中のトラブルの可能性を低く抑えられる可能性があります。

しかし、その安全性に関しては、現在までに十分な検討がされておらず、データは不足しています。

　本研究の目的は、腫瘍径２cmを超える局所進行子宮頸癌（FIGO進行期IB2〜IB3期（2018年分類）の扁平上皮癌、腺癌、腺扁平上皮癌）に対してNAC後に子宮頸部円錐切除術および腹腔鏡下骨盤リンパ節郭清術による妊孕性温存を行い、その腫瘍学的安全性(癌の治療として十分安全であるか)を検討することです。

**４．この研究の方法、期間**

**１）　研究の方法**

当院で子宮頸癌（FIGO進行期IB2〜IB3期（2018年分類）の扁平上皮癌、腺癌、腺扁平上皮癌）と診断されている患者さんのうち、本研究への参加に同意いただける方を対象とします。その他にもいくつか参加いただくための条件があります。詳しくは、後述の「５．研究対象者として選定された理由」をご確認ください。

本研究は以下の流れで実施します。

**＜研究の流れ＞**

**（前観察期間 [スクリーニング期間］）**

研究参加に同意いただいた方には、まずスクリーニングといって、臨床情報（年齢、病名など）の確認を行います。また事前に身体診察（内診、直腸診、コルポスコピー検査、経腟超音波検査）、MRI検査、PET-CT検査（CT検査）も行い、研究への正式な参加に必要な条件をすべて満たしているかどうかの確認をいたします。また、抗がん剤によって卵巣機能が障害され、場合によっては排卵しにくくなる可能性があります。ご希望の場合には、卵子凍結保存、受精卵凍結保存などで対応いたしますのでご相談ください。

上記の確認ができ次第、参加登録をさせていただきます。

**（試験治療（腫瘍径２cmを超える局所進行子宮頸癌に対するNAC後の子宮頸部円錐切除術および腹腔鏡下骨盤リンパ節郭清術による妊孕性温存療法）実施中「介入期間」）**

介入期間には次のような流れで試験治療を行います。まず、NACとしてdose dense TC療法を行います。1回の介入（1サイクル）は3週間かけて行います。1日目に2種類（パクリタキセルとカルボプラチン）を点滴で投与し、8日目と15日目にはパクリタキセルのみを投与します。合計で3サイクル（約9週間）を行います。各サイクル毎に造影MRI検査を行います。３サイクル終了後には全身検索目的にPET-CT検査（または造影CT検査）を行います。次に、子宮頸部円錐切除術を行います。この手術ができるかどうかは、術前の画像検査結果で、癌の大きさが2cm以下、子宮の奥までの浸潤が浅い（半分未満）、他の場所に新たな癌が見つかっていないことが条件になります。手術後の病理検査で、取り残しや再評価が必要な場合には再度子宮頸部円錐切除術をすることがあります。子宮頸部円錐切除術で切除した組織を病理学的に詳しく調べ、切除した癌が2cm以下、子宮の奥深くまでの浸潤が10mm以下、切除したところに癌の取り残しがないことが確認できた場合に、腹腔鏡下骨盤リンパ節郭清術を行い、病理学的に骨盤リンパ節転移がないことを確認します。

本研究による介入中に重い副作用が出た場合には、研究への参加が中止となる場合があります。また、NACの効果が不十分と判断した場合、子宮頸部円錐切除術の摘出物病理検査で安全に子宮を温存することが難しいと判断した場合、リンパ節郭清術で転移が判明した場合などには、子宮全摘術や放射線治療をお勧めする場合があります。あなたの体が過度の再発リスクにさらされることを防ぐために非常に重要なことですので、ご了承ください。

**（後観察期間）**

　病理学的リンパ節郭清陰性を確認してから2年間、病気の再発がないか慎重に経過観察をします。また、QOLに関する質問表にお答えいただきます。

**スケジュール表**

| 項目 | 前観察期間 | 介入  開始日 | 介入期間 | | | | | 後観察期間 |
| --- | --- | --- | --- | --- | --- | --- | --- | --- |
| 時期 | 2～4週前 | 0週 | ddTC^＊１^  1サイクル後 | ddTC  2サイクル後 | ddTC  3サイクル後 | 子宮頸部円錐切除術後 | 腹腔鏡下リンパ節郭清術後 | 終了(中止) |
| 同意取得 | ● |  |  |  |  |  |  |  |
| 患者背景情報の確認 | ● |  |  |  |  |  |  |  |
| 介入期間 |  |  |  |  |  |  |  |  |
| 身体所見 | ● |  | ● | ● | ● |  |  |  |
| 診察 | ● |  | ● | ● | ● |  |  |  |
| 造影MRI | ●^＊2^ |  | ● | ● | ● |  |  |  |
| PET-CT | ●^＊2^ |  |  |  | ●^＊3^ |  |  |  |
| 病理組織検査 |  |  |  |  |  | ● |  |  |
| 病理組織検査 |  |  |  |  |  |  | ● |  |
| 造影CT | ●^＊2^ |  |  |  | ●^＊3^ |  |  | ● |
| 腟部細胞診 |  |  |  |  |  |  |  | ● |
| 質問表 | ● | ● | ● |  |  |  | ● | ● |
| 月経、排卵の有無 |  |  |  |  |  |  |  | ● |

＊1 dose dense TC療法

＊2 スクリーニング検査のうち造影MRI、PET-CT（または造影CT）については同意前１ヶ月以内の情報を用いてもかまいません。

＊3 ddTC３サイクル後の全身検索を目的とした画像検査はPET-CT・造影CT検査のどちらでもかまいません。

**２）　予定参加期間**

この研究は、厚生労働省が設置している公開データベース（通称「jRCT」^＊^）公表日から2030年3月31日まで行われます。

それぞれの患者さんにご参加いただく期間は、前観察期間４週間、介入期間２２

週間、後観察期間104週間（2年間）の約2年6ヶ月となります。

＊jRCTとは、臨床研究等提出・公開システム（Japan Registry of Clinical Trials）のことであり、この研究の情報を登録し、公表しています。

　https://jrct.mhlw.go.jp/

**３）　本研究で使用するお薬について**

この研究では、次のお薬を使用します。

パクリタキセル

カルボプラチン

いずれも、既に子宮頸癌の治療に標準的に使われているお薬です。

**４）　予定参加人数について**

　当院では、試験治療実施例として10例を予定しています。

**５）　研究への参加を中止させていただく場合について**

次のような場合、あなたが研究に同意された場合でも研究を中止させていただくことがあります。また、中止する場合は、その理由およびそれまでのデータの活用方法などを研究担当医師からご説明いたします。

なお、中止した後もあなたと相談しながら最善の治療を行います。中止した場合でも、その後のあなたの体調については必要な限り継続して観察を行います。

1. 本研究に使用する薬剤の品質、安全性、有効性に関する重大な情報が得られた場合
2. もともとあった病気（合併症）が悪くなって、この研究に続けて参加するのが難しいと考えられた場合
3. この研究を始めた後に、参加継続に影響を及ぼすと考えられる副作用等が発生した場合
4. 何らかの理由で、この研究全体が中止となった場合
5. あなたが「4.6）研究に参加された場合に守っていただきたいこと」を故意に守らなかった場合
6. その他、研究担当医師が研究の中止が適当であると判断した場合

**６）　研究に参加された場合に守っていただきたいこと**

現在、あなたが他の病院に通院されている場合は、その病院と病名、使用しているお薬をお知らせ下さい。また、薬局等で購入して使用しているお薬がある場合もお知らせ下さい。これらは、研究を安全に行うために大切なことです。また、あなたが他の病院に通院されている場合は、この研究に参加していることをその病院にお知らせすることがありますので、ご了承下さい。

**７）　新しい情報を入手した場合**

研究期間中に、あなたの安全性や研究への参加の意思に影響を与えるような新たな情報が得られた場合には、すみやかにお伝えします。続けてこの研究に参加されるかどうかは、あなたの自由意思でお決めください。

**５．研究対象者として選定された理由**

この研究に参加される患者さんは、下記の基準に沿った方が選定されます。

**１）　選択基準（研究にご参加可能な方の主な条件）**

1. 画像検査や内診で子宮頸癌ⅠB2あるいはⅠB3期（FIGO2018年分類）

と診断された方

1. 病理組織学的に診断の確定した扁平上皮癌、腺癌、腺扁平上皮癌の方
2. 未閉経の方
3. 同意取得時点で40歳未満の方
4. この研究を安全に受けられる体の状態（血液検査や心電図などで臓器の働きに問題がない）の方
5. 妊孕性温存を希望される方
6. 本研究の参加にあたり十分な説明を受けた後、十分な理解の上、ご本人の自由意思による文書同意をしていただいた18歳以上の方

**２）　除外基準（研究にはご参加できない方の主な条件）**

1. 病理組織学的にHPV非関連性の癌の方
2. 他の癌に罹患している方
3. 重篤な合併症をお持ちの方
4. ポリオキシエチレンヒマシ油（クレモホールEL^R^）含有製剤（シクロスポリンなど）および硬化ヒマシ油含有製剤（注射用ビタミン剤など）の投与歴に関連して過敏症が発現したことのある方
5. 治療が必要な感染症に罹っている方
6. 妊娠、授乳中もしくは妊娠している可能性のある方
7. 未成年者やご自身で同意をすることが難しい方
8. その他、研究責任医師、研究分担医師が対象者として不適当と判断した方

これらの基準に適合している患者さんに、今回研究への参加をお願いしております。

**６．この研究に参加することにより生じる負担、予測されるリスクおよび利益**

**１）　予想される利益**

この研究に参加することで妊孕性温存が可能となるかもしれません。

**２）　予想される不利益**

この研究に参加されますと、以下のような不利益がある可能性があります。この研究を担当する医師は、あなたの健康状態を常に確認し、あなたへの負担が最小限となるよう心がけて研究を行います。

1. 手術による切除範囲の縮小により腫瘍の遺残、あるいは再発のリスクが高まる可能性があります。
2. 術前化学療法による腫瘍縮小効果が得られなかった場合、化学療法中の病勢悪化をきたす可能性があります。
3. 脱毛、末梢神経障害、卵巣機能障害をはじめとするdose dense TC療法に伴う毒性の可能性、検査回数の増加による検査負担の増大などの可能性もあります。

**３）　予想される副作用/合併症**

この研究で使われるお薬であるパクリタキセル、カルボプラチンは、これまでの研究から以下の副作用が起きることが報告されています。

1. あなたの健康に大きな影響を及ぼす副作用

- パクリタキセル

①ショック（0.2%）、アナフィラキシー（0.3%）

②白血球減少等の骨髄抑制

　白血球減少（61.4%）、好中球減少（55.5%）、ヘモグロビン減少（30.7%）、ヘマトクリット値減少（5.0%）、赤血球減少（11.2%）、血小板減少（11.7%）、汎血球減少など

③末梢神経障害（43.8%）、麻痺（0.1％）

④間質性肺炎（0.5%）、肺線維症（頻度不明）

⑤急性呼吸窮迫症候群（0.1％未満）

⑥心筋梗塞、うっ血性心不全（0.1％未満）、心伝導障害（頻度不明）、肺塞栓（0.1％）、血栓性静脈炎（0.4％）脳卒中、肺水腫（0.1％未満）

⑦難聴（0.2％）、耳鳴（0.5％）

⑧消化管壊死（頻度不明）、消化器穿孔、消化管出血（0.1％未満）、消化管潰瘍（0.1％）

⑨重篤な腸炎

　出血性大腸炎（0.1％未満）、偽膜性大腸炎（頻度不明）、虚血性大腸炎（頻度不明）など

⑩腸管閉塞（1.6％）、腸管麻痺（0.1％）

⑪肝機能障害（4.0％）、黄疸（0.1％未満）

⑫膵炎（0.1％未満）

⑬急性腎障害（0.2％）

⑭中毒性表皮壊死融解症、皮膚粘膜眼症候群（頻度不明）

⑮播種性血管内凝固症候群（0.1％）

⑯腫瘍崩壊症候群（頻度不明）

⑰白質脳症（可逆性後白質脳症症候群を含む）（頻度不明）

- カルボプラチン

①骨髄抑制

　汎血球減少（0.1%未満）、ヘモグロビン減少（40.1%）、赤血球減少（36.1%）、ヘマトクリット値減少（31.7%）、白血球減少（56.4%）、好中球減少（7.4%）、血小板減少（42.7%）、出血（0.1%未満）など

②ショック、アナフィラキシー（0.1%未満）

③間質性肺炎（0.1%）

④急性腎障害（0.1%未満）、ファンコニー症候群（頻度不明）

⑤肝不全、肝機能障害、黄疸（頻度不明）

⑥消化管壊死、消化管穿孔、消化管出血、消化管潰瘍（頻度不明）

⑦出血性腸炎、偽膜性大腸炎（頻度不明）

⑧麻痺性イレウス（0.1%未満）

⑨脳梗塞（0.1%未満）、肺梗塞（頻度不明）

⑩血栓・塞栓症（頻度不明）

⑪心筋梗塞、うっ血性心不全（頻度不明）

⑫溶血性尿毒症症候群（頻度不明）

⑬急性呼吸窮迫症候群（頻度不明）

⑭播種性血管内凝固症候群（頻度不明）

⑮急性膵炎（頻度不明）

⑯難聴（0.1%未満）

⑰白質脳症（可逆性後白質脳症症候群を含む）（頻度不明）

⑱腫瘍崩壊症候群（頻度不明）

⑲うっ血乳頭、球後視神経炎、皮質盲（頻度不明）

⑳溶血性貧血（頻度不明）

2.その他の副作用

パクリタキセル、カルボプラチンは、以下の表に示すような、その他の副作用が報告されています。

- パクリタキセル

|  | 20％以上 | 5～20％未満 | 5％未満 | 頻度不明 |
| --- | --- | --- | --- | --- |
| 過敏症^注）^ |  | 発疹 | 発赤 |  |
| 循環器 |  | 低血圧 | 不整脈、頻脈、徐脈、期外収縮、高血圧、心悸亢進、心電図異常、心房細動、心室細動、心肥大、狭心症 |  |
| 消化器 | 悪心・嘔吐（35.1%） | 下痢、食欲不振、口内炎、便秘 | 消化不良、鼓腸放屁、胃炎、腹部膨満感、直腸疼痛、嚥下障害、歯肉炎、直腸障害、口唇炎、舌苔、歯肉痛 | 食道炎、粘膜炎、腹水、腸間膜血栓症 |
| 肝臓 |  | AST上昇、Al-P上昇、LDH上昇、ALT上昇 | ビリルビン上昇 |  |
| 泌尿器 |  | 電解質異常、BUN上昇 | クレアチニン上昇、蛋白尿、排尿困難、血尿、尿失禁、尿閉、出血性膀胱炎 |  |
| 皮膚 | 脱毛（45.3%） |  | そう痒、皮膚疾患、爪の障害、皮膚潰瘍、蕁麻疹、皮膚炎、色素沈着、皮膚乾燥、表皮剥離、皮膚腫脹、爪変色 | 斑状丘疹性皮疹、強皮症様変化、亜急性皮膚エリテマトーデス、手足症候群 |
| 精神神経系 |  |  | めまい、不眠、不安、うつ病、傾眠、思考異常、振戦、失神、激越、神経学的疾患、痙攣、運動失調、健忘症、緊張低下、意識障害、寡動、言語障害、緊張亢進、精神症状、譫妄、眼振、不随意運動、嗄声、気分変動 |  |
| 感覚器 |  |  | 味覚倒錯、味覚喪失、視力異常、眼疾患、結膜炎、耳痛、眼痛、霧視、流涙増加、眼精疲労、飛蚊症、眼乾燥、角膜炎、舌異常感、結膜出血、光視症 | 暗点、黄斑浮腫 |
| 呼吸器 |  | 呼吸困難 | 低酸素症、咳増加、喀痰増加、咽頭不快感 |  |
| 全身症状 |  | 無力症、腹痛、倦怠感、頭痛 | 浮腫、疼痛、インフルエンザ様症候群、腹部腫脹、さむけ、体重増加、体重減少 |  |
| 筋骨格 | 関節痛（32.3%）、筋肉痛（28.8%） | 骨痛、背部痛 | 頸部痛、腰痛 | 筋力低下 |
| その他 |  | 発熱、潮紅 | 胸痛、出血、注射部反応、末梢性浮腫、総蛋白減少、アルブミン減少、骨盤痛、発汗、吃逆、口渇、不正出血、無月経、注射部痛、酩酊感、高血糖、低血糖、脱水 | 血栓症 |

注）投与を中止すること

- カルボプラチン

|  | 10％以上 | 1～10％未満 | 1％未満 | 頻度不明 |
| --- | --- | --- | --- | --- |
| 消化器 | 悪心・嘔吐（50.5％）、食欲不振（45.4％） | 下痢、口内炎、腹痛、便秘 | 口渇 |  |
| 腎臓 |  | 血尿、蛋白尿 | 乏尿 |  |
| 過敏症^注）^ |  | 発疹 | 瘙痒感 | 蕁麻疹 |
| 精神神経系 |  | 末梢神経障害（しびれ等）、頭痛 | 耳鳴、聴力低下、視力障害、眩暈、痙攣、異常感覚、味覚異常、神経過敏、不安、不眠 |  |
| 肝臓 | ALT上昇（10.2%） | A S T 上昇、Al-P上昇、ビリルビン上昇、LDH上昇、γ-GTP上昇 |  |  |
| 循環器 |  |  | 心電図異常（期外収縮）、心悸亢進、血圧上昇、血圧低下、不整脈（頻脈、徐脈、心房細動、心房粗動、房室ブロック） |  |
| 電解質 |  | 血清ナトリウム、カリウム、クロール、カルシウム、リン、マグネシウム等の異常 | 抗利尿ホルモン分泌異常症候群 |  |
| 皮膚 | 脱毛（18.3%） |  | 色素沈着、爪の変色、皮膚疾患 |  |
| その他 | 全身倦怠感（18.6%） | 発熱、浮腫 | 疼痛、潮紅、ほてり、胸部不快感、吃逆、注射部位反応（発赤、腫脹、疼痛、壊死、硬結等）、低蛋白血症 | 無力症、尿酸上昇、悪寒、脱水、体重減少、アルブミン低下、呼吸困難 |

注）投与を中止すること

その他にも、知られていない副作用が起きる可能性もあります。

研究期間中、副作用などの症状が現れていないか注意深く観察していきます。患者さんに副作用が現れた場合は適切な治療を行いますので、いつもと違うと感じる事がありましたらすぐに研究担当医師へお伝えください。

**７．研究への参加自由と同意撤回について**

この研究への参加は、あなたの自由意思によるものです。この研究についてご理解いただき、あなたの自由意思で研究に参加していただける場合は、別紙「臨床研究同意書」に署名をお願いします。一度同意された場合でも、いつでも撤回することができます。その場合は担当の医師に口頭で伝え、別紙「同意撤回書」に署名して下さい。同意を撤回された場合も、原則としてそれまでの情報等は研究に使用させていただきます。これは、研究結果を正しく評価するために必要な方法ですので、ご了承ください。なお、同意されなかったり、同意を撤回されたりしても、それによって診療上不利になることは一切ありません。

**８．研究に関する情報公開の方法**

研究の実施に先立ち、臨床研究等提出・公開システム（jRCT）のデータベースに事前登録をし、研究終了後は結果を公表いたします。また、研究結果は医学の論文や学会などで発表される予定です。

**９．研究計画書および研究の方法に関する資料の入手または閲覧について**

この研究に関して、研究計画や関係する資料をお知りになりたい場合は、他の患者さんの個人情報や研究全体に支障となる事項以外はお知らせすることができます。ご希望がございましたら研究担当医師にお申し出ください。

**１０．個人情報の取扱い**

カルテから抽出した情報等の管理は研究独自のコード番号等で行い、あなたの名前などの情報が第三者にはわからないように十分配慮いたします。

この研究で得られた結果は、医学雑誌や学会などで公表しますが、あなたの名前などの情報は一切わからないようにしますので、プライバシーは守られます。

また、この研究で得られた情報が、本研究の目的以外に使用されることはありません。

また、この研究で得られた情報を将来の研究のために使用する場合は、新たに倫理委員会の判断を仰ぎます。この場合も、あなたのプライバシーが守られるように十分配慮いたします。

**１１．試料・情報の保管及び廃棄の方法**

研究で得られた情報は、本研究のために使用され、研究の終了について報告された日から5年を経過した日までの期間、岡山大学病院産科婦人科の医局で鍵のかかる場所において保管させていただきます。その後シュレッダーにかけ、ファイルを削除し、完全に廃棄します。本研究で提供いただいた試料（血液や組織）は、岡山大学病院内のバイオバンク（試料保管施設）において、適切な管理体制のもと、厳重に保管されます。保管された試料は、将来の医学研究に活用される可能性がありますが、その際にはすべて個人が特定されないよう匿名化され、倫理審査委員会の承認を受けた研究に限り使用されます。ご本人から研究への同意の撤回があった場合、未使用の試料はすみやかに廃棄されます（すでに研究に使用された試料やデータについては取り消すことができない場合があります）。また、保管期間が終了した後や、研究の目的が達成された場合にも、適切な手順に従い廃棄されます。廃棄は、再利用や情報漏洩が起こらないように処理されますのでご安心ください。

**１２．研究資金及び利益相反について**

この研究は、岡山大学病院産科婦人科の研究費で実施します。

なお、研究者はあらかじめ利益相反（外部との利益関係により、研究に従事する者としても社会的責任と、外部との関係によって得る利益とが衝突・相反するため、研究者として必要な「公正な姿勢」が損なわれる、または損なわれるのではないかと第三者から疑われること）について、臨床研究審査委員会に申告し、その管理に問題がないことを確認し

ています。また、研究の経過を定期的に臨床研究審査委員会へ報告を行うことにより、この研究の利害関係についての公平性を保ちます。

**１３．経済的負担、謝礼について**

本研究で実施される介入は、すべて通常の保険診療の範囲内で行われます。研究に参加されることにより、特別な検査が追加されることはありません。ただし、本研究で行われる試験治療は、他の標準治療（手術や放射線治療を中心とした治療）と比較して、通院回数や組み合わせにより、若干多くの医療費負担が発生する可能性があります。なお、高額療養費制度などにより、自己負担額には一定の上限が設けられています。

また、研究に参加していただいても、謝礼や交通費などの支給がないことをご了承ください。

**１４．この研究に参加しない場合の、他の治療方法**

あなたと同じ病気の治療には広汎性子宮全摘術や放射線療法などがあります。あなたが今回、この研究に参加されない場合には、この病院で行っている治療法のうちあなたに最も良いと考えられる治療法により治療を行っていくことになります。

**１５．研究終了後の対応**

この研究が終了した後は、この研究で得られた成果も含めて、研究担当医師は責任をもって最も適切と考える医療を提供いたします。

**１６．研究実施に伴う重要な知見が得られる場合に関する取扱い**

研究の実施に伴い、①あなたの健康に関する重要な知見、または②その他の重要な知見が得られた場合は、あなたにお伝えすることがあります。

**１７．健康被害に対する補償について**

この臨床研究は、科学的に計画され慎重に行われますが、この研究への参加中あるいは終了後に、患者さんに使用したパクリタキセル、カルボプラチンによると思われる副作用などの健康被害が生じた場合には、ただちに適切な処置および治療を行います。本研究で使用する薬剤は、すでに市販されている医薬品をその適応内で使用して行いますので、薬剤による健康被害の治療も通常の診療と同様に研究に参加する患者さんの健康保険を用いて行います。手術によって健康被害が生じた場合にも保険診療内で対応します。

なお、今回の研究の実施にあたり、臨床研究保険に加入しており、万が一、研究計画の不備に起因して健康被害が生じた場合には研究責任医師の加入する保険から補償の給付を受けることができます。

**１８．試料・情報の二次利用、他の研究機関への提供について**

研究で得られた試料（血液や組織）や情報は、原則として本研究のために使用され、研究の終了について報告された日から５年間、ただちに個人を識別できない状態にした上で岡山大学病院内のバイオバンク（試料保管施設）にて保管させていただきます。将来、子宮頸癌に関する新たな研究が計画され、今回の研究で得られた試料や情報を医学研究に用いる場合には、改めて研究計画書を提出し、倫理委員会の承認を受け、あなたの同意を得るか、または、情報公開により研究対象者となることを拒否できる機会を設けます。

**１９．モニタリング、監査について**

あなたの人権が守られながら、きちんとこの研究が行われているかを確認するために、この臨床研究の関係者（臨床研究審査委員会、当院の職員、規制当局の担当者、その他統括管理者が指名した者など）があなたのカルテなどの医療記録を見ることがあります。これをモニタリングあるいは監査といいます。しかし、あなたのプライバシーにかかわる情報（住所・氏名・電話番号など）が外部に漏れる心配はありません。また、報告書などでその情報があなたであると特定されることはありません。

なお、今回の研究では監査は行いません。

**２０．知的財産権の帰属について**

この研究から知的財産権などが生じる可能性はありません。

**２１．研究対象者等からの相談への対応について**

この研究について知りたいことや、ご心配なことがありましたら、遠慮なく研究担当医師にご相談下さい。

相談窓口担当者

岡山大学病院産科婦人科　長尾 昌二

　　住所：〒700-8558　岡山市北区鹿田町2-5-1

　　電話番号：086-235-7320（平日：8時～18時）（産科婦人科医局直通）

　　　　　　　086-235-7885（平日夜間、休日）（東５階病棟）

また、岡山大学病院では、下記の苦情・相談窓口を設けております。

岡山大学病院総合患者支援センター　治験・臨床研究相談窓口

連絡先：086-235-7744（平日8:30～17:00）

E-mail: [iscps@okayama-u.ac.jp](mailto:iscps@okayama-u.ac.jp)

臨床研究同意書

岡山大学病院長　殿

研究責任医師　長尾昌ニ　殿

　私（患者）は貴院における「妊孕性温存を希望する子宮頸癌IB2-IB3期に対する主治療前化学療法を用いた縮小手術」の臨床研究に協力するにあたり、下記について十分に説明を受け、納得した上で臨床研究に参加することを同意します。

記

1. 臨床研究の名称及び当該研究の実施について、実施医療機関の管理者の承認を受けている旨及び厚生労働大臣に実施計画の提出を行っている旨
2. 統括管理者の氏名又は名称、研究責任医師の氏名及び職名並びに実施医療機関の名称（他の実施医療機関と共同して研究を実施する場合には、共同実施医療機関の名称及び共同実施医療機関の研究責任者の氏名及び職名を含む）
3. 臨床研究の対象者として選定された理由
4. 当該臨床研究の実施により予期される効果及び危険
5. 臨床研究の参加を拒否することは任意であること
6. 同意の撤回に関する事項
7. 臨床研究の参加を拒否すること又は同意を撤回することにより不利益な取扱いを受けないこと
8. 臨床研究に関する情報公開の方法
9. 臨床研究の対象者又はその代諾者の求めに応じて、他の臨床研究の対象者等の個人情報等の保護及び当該研究の独創性の確保に支障がない範囲内で研究計画書及び研究の方法に関する資料を入手又は閲覧できる旨並びにその入手又は閲覧の方法
10. 当該臨床研究の対象者の個人情報の保護に関する事項
11. 試料・情報の保管及び廃棄の方法
12. 臨床研究に用いる医薬品等の製造販売をし、又はしようとする医薬品の製造販売業者及びその特殊関係者の当該臨床研究に対する関与に関する状況
13. 苦情及び問い合わせへの対応に関する体制
14. 当該臨床研究の実施に係る費用に関する事項
15. 他の治療法の有無、内容、他の治療法により予期される効果及び危険との比較
16. 当該臨床研究の実施による健康被害に対する補償及び医療の提供に関する事項
17. 当該臨床研究の適否等について審査を行う臨床研究審査委員会における審査事項その他当該臨床研究に係る臨床研究審査委員会に関する事項
18. その他臨床研究の実施に関し必要な事項

　説明日：西暦　　　　年　　月　　日

　説明者（研究責任医師又は研究分担医師名）：　　　　　　　　　科　署名：

【本人（研究対象者）】

同意年月日：西暦　 　年　　月　　日　署名：

＊研究責任医師（分担医師）は記載漏れのないことを確認した後、説明日、説明者欄を記入し、一部を研究者が原本として保管し、複写を**同意説明文書と共に**同意者に交付する。

臨床研究同意撤回書

岡山大学病院長　殿

研究責任医師　長尾昌ニ殿

　私（患者）は貴院における「妊孕性温存を希望する子宮頸癌IB2-IB3期に対する主治療前化学療法を用いた縮小手術」の臨床研究への参加に同意し、「臨床研究同意書」に署名しましたが、その同意を撤回することを研究担当医師に伝え、ここに同意撤回書を提出します。

同意撤回書受領者（研究責任医師又は研究分担医師名）：　　　　　　　　科　　　　　　　　　　　署名：

【本人】

同意撤回年月日：西暦　 　年　　月　　日

署名：

【代諾者】

同意撤回年月日：西暦　 　年　　月　　日　署名：

（研究対象者との続柄　　　　　　　　研究対象者名　　　　　　　　　　　　　　）

＊研究責任医師（研究分担医師）は記載漏れのないことを確認した後、説明日、説明者欄を記入し、一部を研究者が原本として保管し、複写を同意撤回者に交付する。
