# Supplementary material for: Fertility-sparing surgery with neoadjuvant chemotherapy in early and locally advanced cervical cancer: A clinical protocol
Source: PLoS One. 2026 Jan 13;21(1):e0340963. doi: 10.1371/journal.pone.0340963 (PMC12798975; doi:10.1371/journal.pone.0340963)
Supplement: S5 File — (DOCX) [file pone.0340963.s005.docx]

# Explanation and Consent Form for Clinical Research

## To Patients

You are being invited to participate in a clinical research study entitled “Fertility preservation using neoadjuvant chemotherapy and cervical conization followed by laparoscopic pelvic lymphadenectomy for FIGO stage IB2–IB3 cervical cancer.”

This document provides an explanation of the study. Please read it carefully and discuss it with your family and physicians before deciding whether to participate.

## Introduction

This clinical research aims to investigate a treatment strategy that preserves fertility in patients with locally advanced cervical cancer (FIGO stage IB2–IB3) who wish to have children in the future. Standard treatment for this stage of cervical cancer is radical hysterectomy, which results in permanent loss of fertility. In this study, patients will receive neoadjuvant chemotherapy (NAC) prior to the main treatment, followed by a less invasive surgical procedure (cervical conization and laparoscopic pelvic lymphadenectomy). By evaluating the safety and feasibility of this approach, the study seeks to establish a new treatment option for young women with cervical cancer.

## Research Organization

This study will be conducted under the responsibility of the principal investigator and a team of collaborating physicians at Okayama University Hospital. The research has been reviewed and approved by the Certified Review Board (CRB) of Okayama University Hospital.

## Background and Purpose of the Study

Cervical cancer is one of the most common gynecological malignancies, particularly among young women of reproductive age. For patients with FIGO stage IB2–IB3 disease, radical hysterectomy or concurrent chemoradiotherapy is usually performed, but both result in loss of fertility. Fertility preservation is therefore a major unmet need in this patient population.

This study is designed to evaluate the oncological safety and feasibility of a fertility-preserving approach involving neoadjuvant chemotherapy followed by cervical conization and laparoscopic pelvic lymphadenectomy. The goal is to establish an alternative treatment option that maintains both cancer control and the possibility of future pregnancy.

## Study Methods

Eligible patients who provide informed consent will first receive three cycles of neoadjuvant chemotherapy (dose-dense TC therapy: paclitaxel and carboplatin). After completion of chemotherapy, the tumor size will be re-evaluated using imaging and clinical examinations.

If the tumor is reduced to ≤2 cm in diameter and no new lesions are observed, patients will undergo cervical conization. Pathological evaluation of the excised tissue will then be performed. If the surgical margins are negative and the depth of stromal invasion is within acceptable limits, laparoscopic pelvic lymphadenectomy will follow.

If residual tumor >2 cm or positive lymph node metastasis is detected, standard treatment such as radical hysterectomy or concurrent chemoradiotherapy will be performed instead of fertility-preserving surgery.

All patients will be followed for two years after treatment to assess both oncological safety and fertility outcomes. Gynecological assessments, imaging studies, and quality of life surveys will be conducted periodically during the follow-up period.

## Study Participants

Participants in this study are patients diagnosed with cervical cancer (FIGO stage IB2–IB3, 2018 classification) who wish to preserve their fertility. Patients must meet the following conditions:

- Histologically confirmed squamous cell carcinoma, adenocarcinoma, or adenosquamous carcinoma
- Age 40 years or younger
- Premenopausal status
- Good general condition with sufficient organ function
- Strong desire to preserve fertility
- Voluntary written informed consent after receiving adequate explanation about the study

Patients with the following conditions will not be eligible to participate in the study:

- HPV-independent carcinoma
- Presence of other active malignancies
- Severe complications or serious medical conditions
- Hypersensitivity to drugs used in this study (paclitaxel or carboplatin)
- Active infections requiring antibiotics
- Pregnant or breastfeeding women
- Any condition judged by the investigator to make participation inappropriate

## Risks and Benefits

Participation in this study involves certain risks. The investigational treatment includes chemotherapy and surgical procedures, both of which may cause side effects or complications.

### Possible Risks

1. Side effects of chemotherapy (paclitaxel and carboplatin):
- Nausea, vomiting, loss of appetite
- Hair loss
- Bone marrow suppression (low white blood cell count, anemia, low platelet count), which may increase the risk of infection or bleeding
- Peripheral neuropathy (numbness or tingling in the hands and feet)
- Allergic reactions

2. Risks of cervical conization:
- Vaginal bleeding, infection
- Cervical stenosis
- Risk of miscarriage or preterm birth in future pregnancies

3. Risks of laparoscopic pelvic lymphadenectomy:
- Intraoperative bleeding, organ injury
- Lymphocele (accumulation of lymphatic fluid)
- Lower limb edema
- Infection

4. Other possible risks:
- There is a possibility that the intended fertility preservation may not be achieved due to disease progression or treatment-related factors.

### Possible Benefits

The potential benefit of participating in this study is the preservation of fertility while maintaining oncological safety. If successful, this treatment approach may allow future pregnancy and childbirth, which are not possible with standard radical hysterectomy. However, it is important to understand that fertility preservation and favorable oncological outcomes cannot be guaranteed.

## Voluntary Participation and Withdrawal

Participation in this clinical research is entirely voluntary. You are free to decide whether or not to participate after carefully considering the explanation provided. Even after you have agreed to participate, you may withdraw your consent and discontinue participation at any time, without giving a reason.

If you choose not to participate or if you decide to withdraw after participation, you will not suffer any disadvantages or negative consequences regarding your future medical care at this hospital.

In certain situations, the principal investigator may decide to discontinue your participation in the study for safety reasons or if continuing is deemed inappropriate. In such cases, you will be informed of the reason and provided with appropriate care.

## Protection of Personal Information

Your personal information will be strictly protected in this study. The collected data will be anonymized by assigning a study identification code, and documents linking your name and identification code will be securely stored by the principal investigator. Only authorized research staff will have access to this information.

When the study results are published at academic conferences or in medical journals, no personally identifiable information will be disclosed. All reports will be presented in a manner that ensures complete confidentiality.

The data obtained in this study may be used for future research related to cervical cancer and fertility preservation, but only after approval by the relevant ethics committee. In such cases, the same confidentiality protections will apply.

## Publication of Research Results

The results of this study may be published at academic conferences or in scientific journals. In such publications, no personally identifiable information will be disclosed, and data will be presented only in an anonymized form.

## Costs and Compensation

There are no additional costs to you for participating in this study beyond the usual medical expenses for your treatment. Standard treatment costs, including those for hospitalization, examinations, surgery, and medication, will be your responsibility as with regular care.

In the event of health damage directly caused by participation in this study, appropriate medical treatment will be provided. Compensation for such health damage will be determined in accordance with the rules of Okayama University Hospital.

## Contact Information

If you have any questions about this study, please contact the research team at the following address:

Principal Investigator: Shoji Nagao, MD, PhD
Department of Obstetrics and Gynecology
Okayama University Hospital
2-5-1 Shikata-cho, Kita-ku, Okayama 700-8558, Japan
Tel: +81-86-235-7320

## Consent Form

I have received and understood the explanation of the clinical research entitled “Fertility preservation using neoadjuvant chemotherapy and cervical conization followed by laparoscopic pelvic lymphadenectomy for FIGO stage IB2–IB3 cervical cancer.” I have had the opportunity to ask questions and have received satisfactory answers. I voluntarily agree to participate in this research.

Date: __________________________

Patient’s Name (Signature): __________________________

Patient’s Name (Print): __________________________

Person Explaining the Study (Signature): __________________________

Name of Explainer (Print): __________________________

Date of Explanation: __________________________
