# Supplementary material for: Fertility-sparing surgery with neoadjuvant chemotherapy in early and locally advanced cervical cancer: A clinical protocol
Source: PLoS One. 2026 Jan 13;21(1):e0340963. doi: 10.1371/journal.pone.0340963 (PMC12798975; doi:10.1371/journal.pone.0340963)
Supplement: Attachment — Submitted filename: 20251110PLOS One Response.docx [file pone.0340963.s006.docx]

PONE-D-25-48071

Fertility-sparing surgery with neoadjuvant chemotherapy in early and locally advanced cervical cancer: A clinical protocol

PLOS ONE

Response to Reviewers

We sincerely thank the Academic Editor and all reviewers for their thoughtful and constructive comments on our manuscript entitled “Fertility-sparing surgery with neoadjuvant chemotherapy in early and locally advanced cervical cancer: A clinical protocol.” We have carefully revised the manuscript in accordance with the feedback provided. A detailed, point-by-point response is presented below. All suggested changes have been incorporated into the revised version, and grammatical and formatting corrections have been made throughout.

**Major Comments:**

**Relation to recent GOG studies:**
Two recent GOG phase II trials (Gynecol Oncol. 2025;195:50–58, 59–65) have explored fertility-sparing strategies combining conization and lymphadenectomy. These should be cited and discussed to contextualize the study design and highlight differences or innovations in your protocol.

Response: Thank you for your very helpful comments. The GOG278 trial examined the oncological adequacy, contribution to physical function and quality of life, and perinatal prognosis of simple hysterectomy or cone biopsy plus pelvic lymphadenectomy for early stage IA1-IB1 cervical cancer. It was shown that cone biopsy plus pelvic lymphadenectomy can achieve a reasonable oncological prognosis while ensuring a favorable perinatal prognosis. Our study focused on patients with stage IB2 or IB3 disease whose tumor diameter was reduced to ≤2 cm following dose-dense TC therapy, representing a different patient population. Although the clinical implications of achieving a tumor diameter ≤2 cm through chemotherapy differ from those of patients with an initial tumor diameter ≤2 cm, we consider the GOG-278 data to provide supporting evidence for the validity of our approach.

We added some discussion in "Introduction" (line 49 to 57) and "Results and Discussion" section (line 238 to 241).

**Dual experimental approach:**
The concurrent use of NAC and fertility-sparing surgery represents a “double experimental” design. The rationale for combining these interventions should be explicitly justified, and the potential risks of this approach discussed.

Response: As you pointed out, we acknowledge the risks associated with the double experimental approach. Based on the results of SHAPE, ConCerv, and GOG-278, we believe that the oncological safety of less radical surgery for stage IA-IB1 cervical cancer, particularly conization, as investigated in the ConCerv and GOG-278 trials, has been confirmed to be oncologically safe to a certain extent, although the evidence remains limited when conization alone is considered. Furthermore, the SGSG016 study confirmed a high response rate of over 90% and a low PD rate for dose-dense TC chemotherapy, confirming that it is a suitable regimen for preoperative tumor reduction. As described above, we believe that the risks associated with the double experimental approach are within an acceptable range as long as measures are taken to adequately ensure participant safety. This study clearly defines methods for ensuring patient safety based on the results of imaging tests during dose-dense TC therapy and before conization, the histopathological results of the conization specimens, and the histopathological results of the pelvic lymph node dissection specimens. Furthermore, to address the potential risks to ovarian function caused by chemotherapy, it is recommended that embryo freezing or oocyte freezing be performed before starting dose-dense TC therapy.

We have added some discussion in "Results and Discussion" section (line 263 to 277).

**Oncologic staging:**
The protocol does not include a pre-treatment sentinel lymph node biopsy (SLNB), which is critical for accurate baseline staging. Please justify this omission or consider including SLNB (with ultrastaging) as a criterion for eligibility (pN0, macro- or micrometastasis).

Response: Although sentinel lymph node biopsy (SLNB) with ultrastaging provides accurate nodal assessment, nodal status will be assessed radiologically (PET-CT and MRI) before NAC and confirmed pathologically by laparoscopic complete pelvic lymph node dissection after conization in this protocol. It was not included in this protocol for three reasons: first, it avoids additional invasive procedures before chemotherapy and maintains the feasibility of treatment. Second, SLNB after NAC is not currently standard practice and further increases the experimental approach. Finally, SLNB for early-stage cervical cancer is not currently covered by public insurance in Japan. If SLNB becomes eligible for insurance coverage in Japan in the future, it may be possible to consider SLNB for some eligible patients.

We added the discussion regarding SLNB in "Results and Discussion" section (line 278 to 286).

**Endpoints and limitations:**
The primary endpoint (uterine preservation) is clinically meaningful but should be interpreted within the constraints of a short 2-year follow-up, single-center design, and very small sample size (n=10). These limitations should be emphasized in the Discussion.

Response: As you pointed out, the small sample size, single-center design, and short follow-up period (2 years) inherently limit the generalizability of this feasibility study. However, these limitations are appropriate as an exploratory study aimed at generating pilot data for future multicenter Phase II/III trials. There are uncertainties, such as the extent to which this approach will be accepted by Japanese society, the extent of need, and the extent to which support for assisted reproductive technology is required. We selected this design as a practical and feasible exploratory study.

We added the discussion regarding limitation of this study in "Results and Discussion" section (line 295 to 302).

**Statistical plan:**
Include more detail regarding missing data handling, adjustment for confounders, and justification for the sample size (even if feasibility-based).

Response: Missing data will be handled using the last observation carried forward method for patient-reported outcomes, and information will continue to be collected as much as possible for survival endpoints and pregnancy-related information throughout the study. Because this is an exploratory study, a formal power calculation will not be performed. To ensure feasibility, the target sample size was set at 10 (a uterine preservation rate of 70% or more is considered acceptable). If the number of events permits, multivariate exploratory analysis will be performed using Cox regression.

Detailed description has been added to 7. Statistical analysis in "Patients and Methods" section (line 209 to 215).

**Novelty and contribution:**
The use of dose-dense paclitaxel/carboplatin (dd-TC) NAC prior to fertility-sparing surgery could represent a distinctive contribution. Please emphasize this and clearly state how this approach differs from previous reports.

Response: The authors believe that the lack of success of previous NAC studies is due to the low intensity of chemotherapy, the goal of improving prognosis, and the inclusion of cases in which tumors had spread outside the cervix. Regarding chemotherapy regimens in particular, most previous NAC studies have used tri-weekly regimens, whereas this study employed a more intensive regimen called dode-dense paclitaxel. In fact, in the SGSG016 trial, ddTC therapy achieved a response rate of 92%, including a 36% complete response rate, far higher than conventional tri-weekly regimens. We believe this approach is possible only because of the availability of ddTC, a regimen that can effectively shrink tumors.

The response rate and pathological CR rate have been added to the "Results and Discussion" section (line 257 to 258)to further emphasize the usefulness of ddTC therapy.

**Clinical implications:**
The Discussion should better outline how this feasibility study can inform future multicenter trials and guideline development.

Response: We believe that this study will demonstrate that tumor shrinkage through chemotherapy will enable the introduction of less invasive fertility-sparing surgery and further expand its application. This study will clarify its oncological safety and perinatal prognosis, as well as the type of assisted reproductive technology that is required. Furthermore, we believe that this study will clarify whether this approach is socially acceptable, and, if so, the extent of societal demand for it.

We have added some discussion into "Results and Discussion" section (line 305 to 309 and line 312 to 313).

**Minor Comments:**

Clarify imaging modalities for determining lymph node status (PET-CT vs. CT).

Response: Before chemotherapy, FDG-PET is used, and before surgery, contrast-enhanced CT (or plain CT if there is an allergy) is used to confirm the presence or absence of lymph node metastasis. Lymph node metastasis is suspected when the SUV max on FDG-PET is greater than 2.5, or when the short axis on CT is greater than 10 mm. The decision will be made separately by one imaging specialist and one gynecological oncologist, and if there is no agreement, the final decision will be made by discussion (line 113 to 119, line 143 to 145, and 167 to 172).

Specify how tumor size is measured and reviewed (MRI criteria, double reading).

Response: Similarly, tumor size will be measured by pelvic contrast MRI (or plain pelvic MRI if the patient has an allergy) four times in total before chemotherapy and after each cycle. The tumor size will be measured separately by one imaging specialist and one gynecological oncologist, and if there is a discrepancy, a decision will be made through discussion (line 113 to 119, line 143 to 145, and 167 to 172).

Correct minor grammatical inconsistencies.

Response: Fixed some grammatical errors and inappropriate wording.

"a normal" to "an intact" (line 142)

"next" to "subsequent" (line 154)

"following" to "upon" (line 218)

"end" to "conclude" (line 219)

"the" to "an" (line 326)

Add line numbering for easier reference during revision.

Response: We apologize for having omitted the line numbers in the previous version. They have now been added.
