# Supplementary material for: Fertility-sparing surgery with neoadjuvant chemotherapy in early and locally advanced cervical cancer: A clinical protocol
Source: PLoS One. 2026 Jan 13;21(1):e0340963. doi: 10.1371/journal.pone.0340963 (PMC12798975; doi:10.1371/journal.pone.0340963)
Supplement: Attachment — Submitted filename: Response to Reviewers for Manuscript PONE.docx [file pone.0340963.s007.docx]

Response to Reviewers for Manuscript PONE-D-25-48071R1

Manuscript Title: Fertility-sparing surgery with neoadjuvant chemotherapy in early and locally advanced cervical cancer: A clinical protocol.

Response to Reviewers

Comment 1: SLNB: inaccurate and outdated statements

Response: We thank the reviewer for this important and timely comment. At the start of this study (IRB review), only Techne® Phytate used in the RI method was approved in Japan, so sentinel lymph node biopsy was not included in the study. As you pointed out, indocyanine green (ICG), a fluorescent dye tracer, was already approved in 2025, and we have heard that the lymph node biopsy procedure is expected to be covered by insurance in 2026. Actually, our facility introduced sentinel lymph node biopsy into clinical practice in December 2025. The revised manuscript now states the latest information on SNL mapping (Line 263 to 265).

Comment 2: Redundancy between Introduction and Discussion

Response: We appreciate this helpful suggestion. We have substantially streamlined the Discussion by removing repeated background information regarding tumors ≤2 cm and dd-TC chemotherapy. The Discussion now focuses on interpretation of the protocol design, safety considerations, and applicability to IB2–IB3 disease, while detailed background evidence is retained in the Introduction (Line 58 to 64 and 81 to 84).

Comment 3: Dual experimental approach

Response: We agree that explicit interpretation is essential. The Discussion has been revised to clearly describe the specific risks associated with combining neoadjuvant chemotherapy and fertility-sparing surgery, the predefined radiologic and pathological safety criteria, and the thresholds at which fertility preservation is abandoned in favor of standard treatment (Line 252 to 259).

Comment 4: Limitations section

Response: We have added a specific limitation acknowledging the lack of pathological nodal staging prior to neoadjuvant chemotherapy and discussed the potential for residual occult nodal disease despite radiologic response. This point is now clearly stated in the Discussion section (Line 265 to 270).

Comment 5: Length and focus of the Discussion

Response: The Discussion has been reorganized to emphasize the unique contribution of this feasibility study, its safety framework, and its potential implications for future multicenter trials and guideline development. Narrative background has been reduced accordingly in Discussion section.

We sincerely thank the reviewer for recognizing the strengths of the revised manuscript and for providing constructive guidance that has significantly improved its scientific rigor and clarity.
