# Supplementary material for: Fertility-sparing surgery with neoadjuvant chemotherapy in early and locally advanced cervical cancer: A clinical protocol
Source: PLoS One. 2026 Jan 13;21(1):e0340963. doi: 10.1371/journal.pone.0340963 (PMC12798975; doi:10.1371/journal.pone.0340963)
Supplement: Attachment — Submitted filename: Response to reviewers R3.docx [file pone.0340963.s008.docx]

Response to Reviewers for Manuscript PONE-D-25-48071R2

Manuscript Title: Fertility-sparing surgery with neoadjuvant chemotherapy in early and locally advanced cervical cancer: A clinical protocol.

Response to Reviewers

Comment 1: Interpretation of the primary endpoint

The use of uterine preservation as the primary endpoint is appropriate for a feasibility study. However, a brief clarification of how this endpoint should be interpreted alongside oncologic outcomes (e.g., short-term RFS) would improve clinical interpretability. Suggested action: Add one or two sentences in the Discussion clarifying that uterine preservation is evaluated in conjunction with oncologic safety endpoints, rather than as an isolated success measure.

Response: We agree with the reviewer’s suggestion. While the primary goal is feasibility as measured by the uterine preservation rate, this is only clinically meaningful when achieved without compromising oncologic safety. We have added a sentence to the Discussion to clarify this integrated interpretation. We have added the sentence " Importantly, successful uterine preservation in this study is not viewed as an isolated metric but is evaluated in close conjunction with oncological safety endpoints, such as 2-year RFS, to ensure that fertility-sparing does not jeopardize patient survival." to the Results and Discussion section (Line 264 to 267).

Comment 2: Imaging-based assessment after neoadjuvant chemotherapy

The manuscript appropriately explains the rationale for relying on post-NAC imaging and subsequent pelvic lymphadenectomy. For balance, it would be helpful to briefly acknowledge the inherent limitations of imaging-based response assessment, including the possibility of occult residual disease. Suggested action: Add a short statement in the Discussion noting this limitation.

Response: We acknowledge the inherent limitations of imaging-based response assessment as pointed out by the reviewer. We have added a statement in the Discussion noting the possibility of occult residual disease that may not be captured by imaging alone. WE have added the sentence " However, we recognize the inherent limitations of imaging-based assessment, as PET-CT and MRI may not fully detect occult micro-residual disease or small-volume nodal involvement after NAC." to the Results and Discussion section (Line 271 to 273).

Comment 3: Sample size justification

The feasibility nature of this study and the small sample size are acceptable. A short explanation of the practical or ethical considerations that informed the choice of a target sample size of 10 patients would enhance transparency. Suggested action: Provide a brief clarification in the Sample Size or Discussion section.

Response: Thank you for this suggestion. The target sample size of 10 was determined based on the practical challenges of recruiting eligible patients at a single institution and the ethical imperative to limit exposure to this novel dual-experimental strategy until preliminary safety is confirmed. We have clarified this in the Sample Size section. We have replaced the sentence " To ensure feasibility, the target sample size was set at 10 participants, assuming that a uterine preservation rate of ≥70% would be considered clinically acceptable." to " The target sample size of 10 was chosen based on practical considerations regarding the rarity of FIGO stage IB2–IB3 patients seeking fertility preservation at a single center, as well as the ethical necessity of limiting participant exposure to this novel approach until the planned interim safety analysis is completed." at the Sample Size section (Line 220 to 223).

Comment 4: Ethical transparency

Given the dual experimental nature of the proposed strategy, the ethical framework is generally well described. A brief statement emphasizing that patients are explicitly informed of the non-standard and exploratory aspects of this approach would further strengthen the protocol. Suggested action: Add a single sentence to the Ethical Considerations section.

Response: As suggested, we have added a sentence to the Ethical Considerations section to emphasize that all participants are explicitly informed of the non-standard and exploratory nature of the proposed strategy during the informed consent process. We have added the sentence " During the informed consent process, it is explicitly emphasized to all participants that this treatment strategy is non-standard and exploratory in nature, involving potential risks associated with the dual-experimental approach." to the Ethical Considerations section (Line 236 to 239).
